# Hyperuniform Mesoporous Gold Films Coated with Halogen-Bonding Metal–Organic Frameworks for Selective Raman Sensing of Chlorinated Hydrocarbons

**DOI:** 10.1021/acsnano.5c09431

**Published:** 2025-07-24

**Authors:** Sarah Z. Khairunnisa, Olga Guselnikova, Yunqing Kang, Pavel S. Postnikov, Rashid R. Valiev, Jonathan P. Hill, Nugraha Nugraha, Brian Yuliarto, Yusuke Yamauchi, Joel Henzie

**Affiliations:** † Research Center for Materials Nanoarchitectonics (MANA), 52747 National Institute for Materials Science (NIMS), 1-1 Namiki, Tsukuba, Ibaraki 305-0044, Japan; ‡ Doctoral Program of Nanoscience and Nanotechnology, Graduate School, 89224 Institut Teknologi Bandung, Bandung 40132, Indonesia; § Research Center for Nanoscience and Nanotechnology (RCNN), Institut Teknologi Bandung, Bandung 40132, Indonesia; ∥ Department of Materials Process Engineering, Graduate School of Engineering, Nagoya University, Nagoya 464-8603, Japan; ⊥ Research School of Chemistry and Applied Biomedical Sciences, 65078 Tomsk Polytechnic University, 43A Lenin Avenue, Tomsk 634050, Russian Federation; # School of Chemical Engineering, Australian Institute for Bioengineering and Nanotechnology (AIBN), 1974 The University of Queensland, Brisbane, Queensland 4072, Australia

**Keywords:** plasmonics, hyperuniformity, mesoporous metal, surface-enhanced Raman spectroscopy, chlorinated aromatic hydrocarbons, halogen bonding, metal−organic frameworks

## Abstract

The selective detection of chlorinated aromatic hydrocarbons (CAHs) in environmental samples is challenging due to matrix interference effects. We report a surface-enhanced Raman spectroscopy (SERS) sensor that combines mesoporous Au films with UiO-66-I metal–organic framework (MOF) coatings to achieve the selective detection of CAHs. We show that mesoporous Au films can be considered hyperuniform two-dimensional (2D) materials where long-range correlations and local disorder assist in electromagnetic hotspot formation for SERS. Infiltrating the mesoporous Au films with UiO-66-I serves dual functions critical to sensor performance: First, its iodine-functionalized linkers selectively recruit CAHs from complex matrices through halogen bonding (HaB), concentrating target molecules at SERS hotspots while excluding common interferents. Second, the high refractive index of the MOF enhances light coupling by limiting scattered light, concentrating optical energy on the adsorbed CAHs for SERS enhancement. At optimal MOF thickness, the sensor achieves a detection limit below 1 × 10^–10^ M for 1,4-dichlorobenzene and 4-chlorobiphenyl, surpassing environmental standards by several orders of magnitude. The sensor demonstrates excellent selectivity for CAHs over common interferents, including protein, polycyclic aromatic hydrocarbons, and complex environmental matrices. Furthermore, the sensor maintains performance through multiple adsorption–desorption cycles, enabling reuse. This approach combines reticular chemistry with self-assembled nanostructured metals to achieve both high sensitivity and selectivity in complex environmental samples.

## Graphical abstract

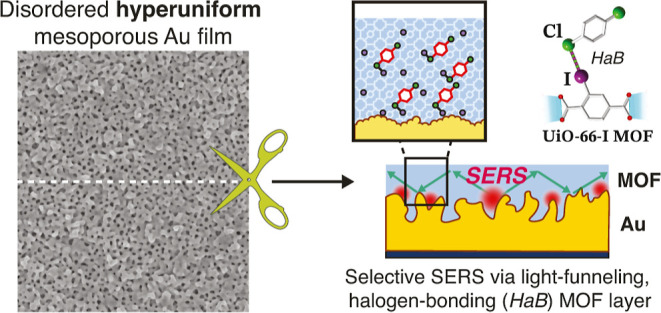

Chlorinated aromatic hydrocarbons (CAHs) are persistent environmental pollutants that originate primarily from industrial processes and agricultural runoff.^1,2^ Selective detection of CAHs in environmental samples is challenging due to complex interference effects within the matrix.^3,4^ Traditional detection methods require sample preparation and complex instrumentation like chromatography and mass spectrometry, which are difficult to deploy in the field.^5^ Surface-enhanced Raman spectroscopy (SERS) is an alternative approach, enabling the rapid optical detection of target molecules with increasingly miniaturized and portable instrumentation.^6^ SERS methods use light to excite collective excitations of free electrons within the gaps and crevices of noble metals called surface plasmons (SPs), which generate highly localized electromagnetic (EM) fields or “EM hotspots” to enhance the Raman vibrations of adjacent molecules.^7–9^ Although SERS enables the trace detection of molecular fingerprints, all molecules within the hotspot are indiscriminately enhanced, making selective detection challenging. The spatial and sample-to-sample reproducibility of EM hotspots is also important for enabling informed decisions when using SERS on environmental and diagnostic samples.

To achieve more selective SERS sensing in complex environments, additional materials are needed to impose molecular selectivity on the plasmonic surface. Researchers have engineered surfaces using alloying,^10^ mesoporous silica,^11^ molecular ligands,^12,13^ metal–organic frameworks (MOFs)^14–17^ and covalent organic frameworks (COFs).^18^ These materials act as intermediaries between molecules and SERS hotspots through intermolecular forces like electrostatic interactions, molecular sieving, hydrogen bonding, π–π stacking, and van der Waals forces. While these materials enhance selectivity by recruiting desired molecules into hotspots, their role in EM enhancement is minimal. MOFs can amplify the Raman cross-section via chemical enhancement, but this contribution is significantly smaller than EM enhancement via the metal.^19^ Hybridizing plasmonic metals with thin-film MOFs combines the advantages of both materials in SERS, and the reticular chemistry approach makes it possible to create MOF films with tunable selectivity toward a target molecular class.^20^ Additionally, MOFs can be engineered with highly optically polarizable linker molecules to amplify and trap optical resonances in the MOF layer, coupling the SP resonances to adsorbed molecules for more selective SERS sensing.^21,22^

In this paper, we describe a selective SERS sensor based on disordered hyperuniform mesoporous gold (mAu) films coated with MOFs for the selective detection of CAHs. Nanostructured films were selected instead of free-standing nanoparticles because films generate superior stability and reproducibility of SERS signals over large areas, making them the preferred choice for commercial SERS substrates.^23^ Incorporating pores in materials creates structural correlations that affect the long-range diffusion of light, even in disordered media.^24^ Near-hyperuniform nanostructures have been investigated for SERS before;^25^ however this pioneering study neither quantified the degree of hyperuniformity nor established a connection between long-range density correlations and EM hotspot formation, leaving ample scope for deeper theoretical and quantitative analysis. We show that the pores formed in the electrodeposition process of mAu films have more uniform spacings than would occur in a random (Poisson) process. The resulting films are disordered hyperuniform systems: long-range correlations enhance coupling of incident light to SPs, while short-range disorder generates nanoscale sites where EM hotspots manifest and amplify the SERS response of molecules, as indicated by experiment and numerical simulations.

The mAu films are infiltrated with a mixed UiO-66-I MOF that has linkers with iodine functional groups. The MOF plays a dual role: (**i**) UiO-66-I bonds with CAHs via the electrophilic σ-hole of the iodine group, forming a halogen bond (HaB).^26,27^ Halogenated MOFs can recruit CAHs from the environment and have been used in chemical separation and remediation of biowarfare agents and pesticides.^28^ (**ii**) The mixed UiO-66-I films have a sufficiently high refractive index (*n*) to play an active role in enhancing SERS by confining light near the surface of the mAu films. The resulting hybrid material increases the adsorption of CAHs *and* absorption of light to enable SERS sensors that are inexpensive to fabricate and can selectively detect CAHs in complex environmental samples.

## Results and Discussion

The mAu films were synthesized by electrodepositing a solution of HAuCl_4_ and monodisperse PS_18,000_-*b*-PEO_7,500_ block copolymer micelles (BCMs) with a polydispersity index (PDI) of 1.09 on a working electrode composed of an Au film on Si wafer (see Section Methods).^29^ The metal electrodeposition process immobilizes the physical arrangement of BCMs, and the resulting metal structure can provide some insight into their assembly mechanism. BCMs are removed by immersing the substrate in tetrahydrofuran (THF) at 40 °C and then dried under nitrogen (N_2_) gas. A scanning electron microscopy (SEM) image of the top surface shows mostly circular pores that are closely spaced (Figure 1a). In the electrodeposition solution, BCMs are highly concentrated, corresponding to ∼128 micelles per μm^3^, or an average center-to-center spacing of roughly 200 nm between micelles (see Note S1 for the calculation). The zeta potential of the BCM solution without HAuCl_4_ is −6.4 mV. Coordination of the Au^3+^ ions to the PEO shell causes the BCM/HAuCl_4_ solutions to become weakly positive at +0.66 mV. The negligible electrostatic repulsion allows the Au-loaded BCMs to pack closely together and assemble based mainly on steric constraints rather than charge, while the hydrated PEO shell helps to resist coalescence with other BCMs, although overlaps are present.

**Figure 1:**
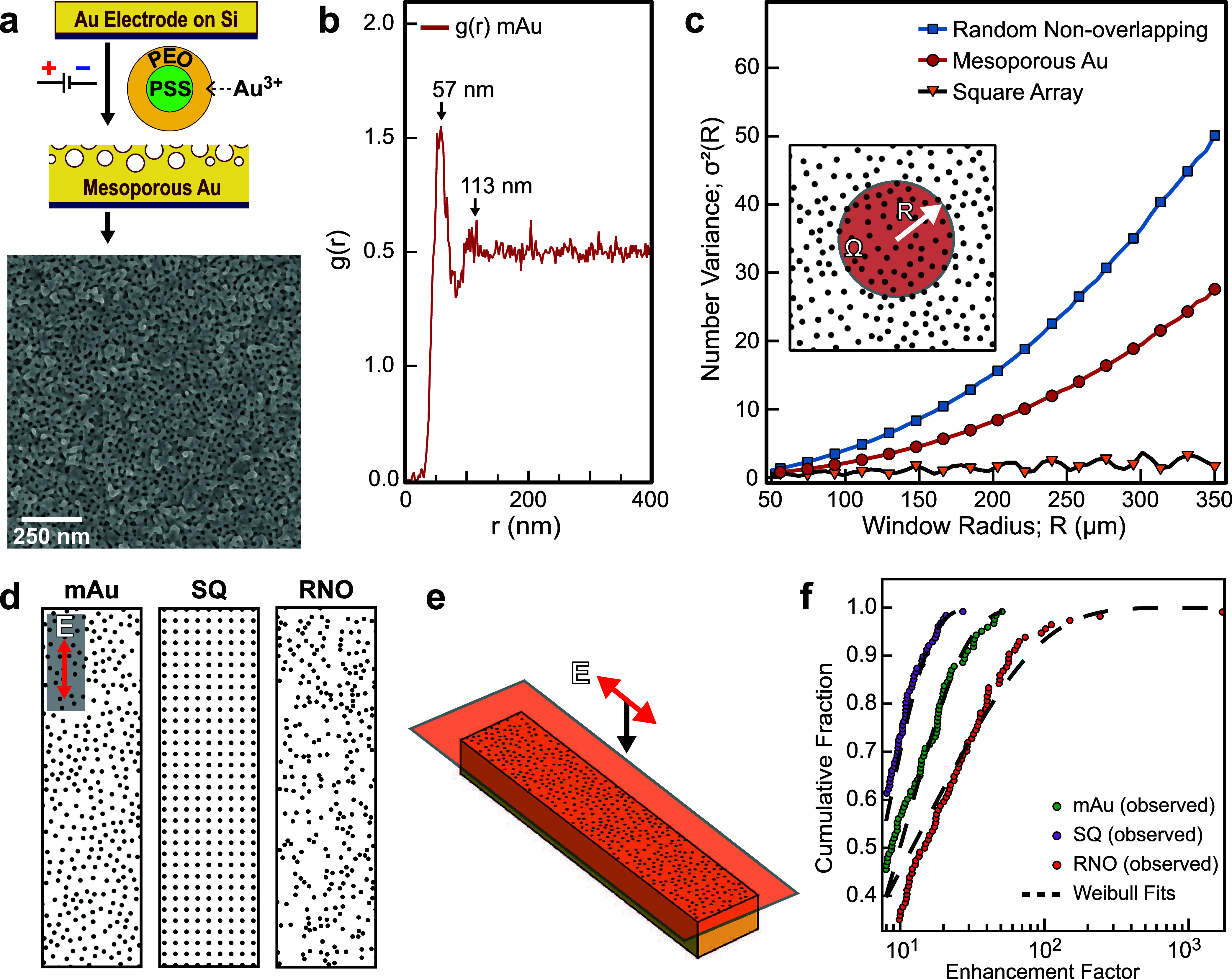
(a) An illustration of the electrodeposition procedure using PSS–PEO block copolymer micelles and gold metal precursor to create mesoporous Au (mAu) on gold electrodes and an accompanying SEM image of the resulting mAu film. (b) Pair-correlation function graph showing the spatial correlations of a representative 3 μm × 3 μm section of the mAu film shown in Figure S1a. (c) Number variance (σ^2^) analysis comparing three patterns: a mAu film, an ordered square array distribution, and a random non-overlapping distribution (Figure S1b,c). The square array exhibits linear oscillating σ^2^ with increasing window radius, demonstrating hyperuniformity characteristic of 2D ordered systems. The mAu pattern exhibits intermediate behavior with variance growing more slowly than random (subquadratic scaling with *R*), indicating disordered hyperuniformity. In contrast, the random non-overlapping pattern displays a steeper scaling of σ^2^ with *R*, approaching quadratic growth typical of nonhyperuniform 2D systems. (d) Three patterns, including hyperuniform mAu, ordered square arrays and disordered random non-overlapping arrays, were converted into nanohole arrays and simulated to examine how morphology affects the frequency of EM hotspots for SERS. (e) The films were excited with a plane wave polarized along the long axis of the films and the electric field intensity maps were monitored in the near field at the top surface of the films as indicated by the transparent red plane. (f) Weibull distributions of the theoretical SERS enhancement factors collected from the E^2^ intensity maps for the three patterns in (d).

A large-scale SEM image of the mAu film was processed with Fiji (ImageJ) and pores were assigned based on contrast variations (Figure S1a). The spatial organization of these pores was quantified using a pair-correlation function (*g*_2_), which reveals how the pores are positioned relative to each other (Figure 1b). This analysis showed a strong correlation between nearest-neighbor pores at 57 nm spacing, with a much weaker second-order correlation at 113 nm. These specific distances emerge from the physical constraints of micelle packing on the electrode surface. Rather than arranging randomly, the micelles organize themselves through steric packing effects, creating relatively consistent spacings between their immediate neighbors. This phenomenon produces more uniform pore distributions compared to a random (Poisson) process, resulting in a reduced variance in spatial distribution which is characteristic of correlated disordered materials.^24^ While the correlation between pores decreases at larger distances, the system appears to maintain long-range uniformity—a fundamental aspect of hyperuniformity, which is a term that describes how effectively a system minimizes density variations across larger length scales.^30^

Hyperuniform materials in many cases can provide desirable optical or mechanical properties while being more tolerant to imperfections, which tend to manifest in self-assembly processes. To assess hyperuniformity, we performed number variance calculations on pore patterns derived from the top surface of mAu films. We also modeled ordered square arrays and disordered patterns of non-overlapping randomly distributed pores (Figures 1c and S1b,c). In hyperuniform systems, the number variance, σ^2^(*R*), within a circular sampling window (Ω) of radius *R* grows more slowly than the window area (∝*R* ^2^; see inset Figures 1c and S1d). Square arrays are characterized as ordered hyperuniform patterns under the hyperuniformity rubric and exhibit linear, oscillating number variance. The window variance of random non-overlapping porous films scale quadratically, exceeding *R* ^2^ as expected for two-dimensional (2D) systems lacking both short- and long-range order. The mAu films exhibit *R* ^1.9^ scaling, classifying them as a disordered type III hyperuniform 2D system according to Torquato’s classification scheme.^31^

EM hotspot frequency at the Raman wavelength (λ = 785 nm) can serve as a proxy for light coupling efficiency that is directly relatable to SERS. To examine the impact of the hyperuniform morphology, we treated the hyperuniform mAu, ordered SQ and disordered RNO patterns as 3.0 μm × 0.6 μm nanohole array supercells with periodic boundary conditions containing roughly the same number of 20 nm diameter cylindrical nanoholes in Au (Figure 1d). The films were excited with a broadband plane wave polarized along the long axis of the films and *E* ^2^ was monitored 3 nm above the top surface of the films at λ = 785 nm to monitor the SERS wavelength (Figure 1e). The 2-parameter Weibull distribution is well suited for capturing the variation in *E* ^2^, which depends sensitively on the nanoscale features of the metal structure in systems with a high degree of complexity.^32^ In the Weibull equation

$$F_{Weibull}\left(x\right)=1-\exp\left\{-{\left(x/b\right)}^{c}\right\}$$

where the *scaling parameter* (*b*) indicates the average *E* ^2^ of a hotspot and the *shape parameter* (*c*) indicates the dispersion of the *E* ^2^ values. Higher *c* indicates lower dispersion in *E* ^2^ (i.e., higher reproducibility). The *E* ^2^ values were squared to obtain the theoretical SERS enhancement factor (*E* ^4^) and then decorrelated to ensure one datum per EM hotspot. EF values above a threshold of 2 were fitted to a Weibull distribution for each pattern (Figure 1f; see complete fit in Figure S2). Table 1 summarizes the parameters obtained from the Weibull distribution. Hyperuniform mAu exhibits a ∼1.8× larger *b* than the ordered SQ, confirming that its short-range disorder couples incident light more efficiently into localized plasmons. At the same time, the *c* of mAu array is ∼1.98× larger than the RNO, indicating a lighter tail and therefore a more even hotspot landscape—an expected consequence of the long-range density uniformity that defines hyperuniformity. Parameter uncertainties (±1σ) were obtained from 2,000 bootstrap resamples, and Kolmogorov–Smirnov tests verified all fits (*p* > 0.05) (Table S1). Taken together, the data place hyperuniform mAu midway between crystalline and random structures, combining strong light coupling with reproducible field localization, which is a desirable balance for SERS substrates in environmental sensing. See the Section Methods for more detailed information on the Weibull analysis.

**Table 1:** Weibull Distribution Fit Parameters

	mAu	SQ	RNO
scale (*b*)	11 ± 1	6.1 ± 0.5	22 ± 4
shape (*c*)	1.07 ± 0.06	1.21 ± 0.08	0.61 ± 0.10

The effect of mesopores on the optical properties of Au films on silicon was examined using absolute optical reflectance (AOR) measurements with an integration sphere setup (Figure 2a). Flat Au films have the characteristic interband transitions <500 nm and rapidly increasing reflectance at longer wavelengths where the free-electron behavior of Au dominates.^33^ The incorporation of pores in the mAu decreases reflectance significantly at NIR wavelengths (e.g., 55% at λ = 785 nm). Mesoporous Au films can be viewed as complex networks of metal junctions, where the optically induced charge transfer can shift the wavelength of the SP depending on junction size and its connectivity with other junctions as the energy dissipates through the metal network (Figure 2b).^34–36^ The hyperuniform metal surface provides additional momentum for light to couple with SPs, while the local nonuniformity caused by the tortuous network of junctions limits propagation and provides points where SPs localize into EM hotspots.

**Figure 2:**
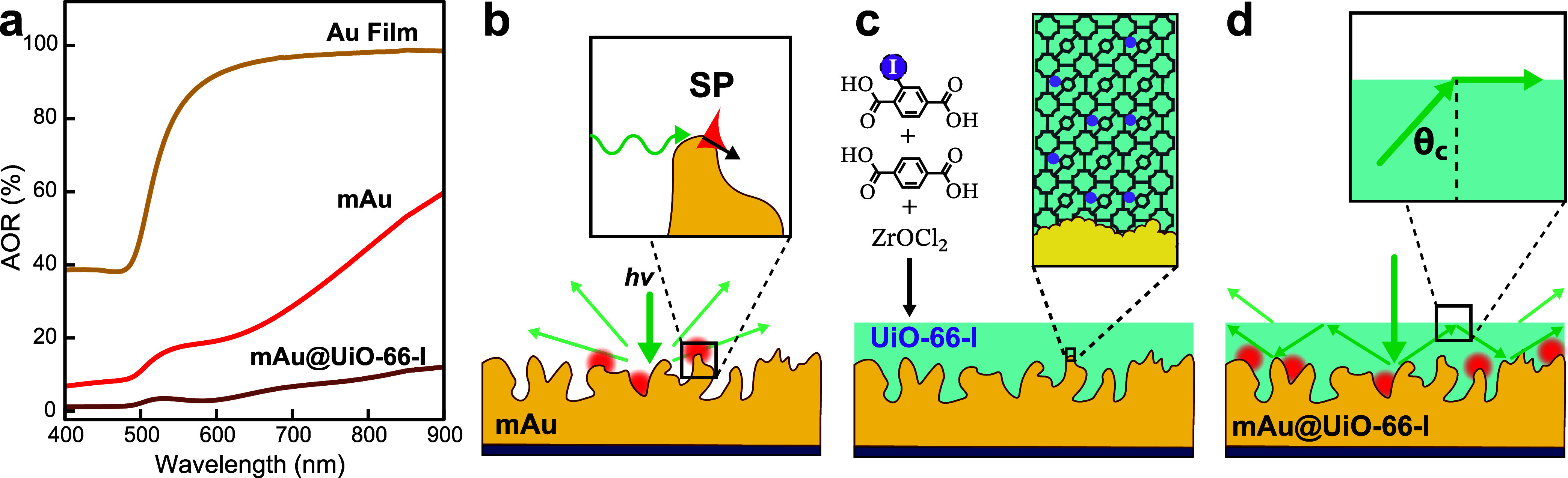
(a) Absolute optical reflectance (AOR) spectra of a flat Au film electrode, a mAu film, and a mAu coated with a 77 nm-thick layer of UiO-66-I MOF. (b) An illustration of the cross-section of a mAu surface showing how light incident on the surface excites SPs. (c) An illustration of the vapor-assisted conversion (VAC) method used to infiltrate and coat the mAu films with a mixed UiO-66-I MOF. (d) When the mAu is coated with a high refractive index dielectric film of sufficient thickness, light propagating within the dielectric and incident at the interface at an angle greater than the critical angle (θ_c_) will undergo total internal reflection (TIR) and remain confined within the film, potentially enhancing the excitation of adsorbed molecules for SERS.

Infiltrating optically active patterns with a material with a higher *n* than the environment is a common strategy to reduce reflectance and further improve coupling with the underlying pattern.^37^ Coating porous plasmonic films with higher *n* dielectric materials may also improve coupling by creating a complex distribution of epsilon near zero (ENZ) regions that form LSPRs with nonuniform electric fields and gradients.^38^ The mAu films were infiltrated with mixed UiO-66-I MOF using a vapor-assisted conversion (VAC) method^39^ in a borosilicate bottle containing ZrOCl_2_, dimethylformamide (DMF), acetic acid, and 50:50 2-iodoterephthalic acid/terephthalic acid to generate the mAu@UiO-66-I films (Figure 2c; see Section Methods). The average thickness (*t*_MOF_) of the MOF film can be varied from 6.4 to 137 nm by changing the precursor concentration, as shown with cross-section SEM images (Figure S3a).

1,4-Dichlorobenzene (DCB) can be used as a model molecule for CAH detection because it contains the key structural features of the class and is a common environmental contaminant with a health-based guidance limit of ∼5 × 10^–7^ M.^40^ The halogen bond (HaB) between DCB and 2-iodoterephthalic acid is estimated to be −1.6 to −2.0 kcal/mol,^27^ enabling it to bond and recruit CAHs from the liquid but is sufficiently reversible to enable the sensor to be potentially regenerated via washing. The mAu and mAu@UiO-66-I films with different *t*_MOF_ were soaked in an aqueous 0.5 mM DCB solution and then monitored at the 1001 cm^–1^ peak of DCB to assess SERS intensity using 785 nm excitation (Figure S3b). SERS intensity increased up to *t*_MOF_ = 77 nm, generating 6× improvement over uncoated mAu films. The theoretical SERS EF for mAu films coated with different thicknesses of MOF was simulated like in Figure 1d–f but monitored normal to the surface to capture the distribution of EM hotspots as they increasingly shift into the dielectric film with a thicker MOF layer. The Weibull distribution indicates that the 77 nm film should generate the strongest SERS EF (Figure S4). Together, the experimental trend and Weibull analysis provide phenomenological support for selecting *t* = 77 nm as the optimal thickness for SERS experiments.

Zirconium-based UiO-66 MOFs can be constructed with ligands achieving *n* up to 1.78, depending on the amount of linker halogenation.^41^ The mAu@UiO-66-I film may enhance light trapping by confining wavevectors internally through total internal reflection (TIR) at the air/UiO-66-I interface for incident angles exceeding the critical angle (θ_c_) (Figure 2d). Extrapolating using data provided by Treger et al.,^41^ UiO-66-I has *n* ∼ 1.468 at an excitation wavelength of 785 nm, thus $\theta_{c}=\sin^{-1}\left(\frac{n_{air}}{n_{MOF}}\right)=42.9^{{}^{\circ}}$ . Light scattered from the mAu surface at angles > θ_c_ may undergo total internal reflection (TIR) at the air/UiO-66-I interface and be redirected back into the mAu film to create more EM hotspots and potentially more SERS signal. The MOF film strongly affects the AOR spectrum of the mAu@UiO-66-I film (*t*_MOF_ = 77 nm) in Figure 2a, decreasing reflectance by ∼82% versus the flat gold film. The weaker reflectance indicates that the MOF film successfully enhances light coupling with the mAu film, which should improve the SERS intensity of adsorbed molecules.

The structure of the pores and infiltration of the MOF was examined using a cross-section of mAu@UiO-66-I created by sputtering the structure with a layer of amorphous carbon (*a*-C) and cutting with a focused ion beam (FIB) and imaged with high-angle annular dark-field scanning transmission electron microscopy (HAADF-STEM) (Figures 3a and S5). Although the zirconium atoms in the UiO-66-I layer have a relatively high atomic number, the density of the MOF is small; thus it appears as a darker 67 nm thick layer sandwiched in between the mAu and the sputtered *a*-C film (inset Figure 3a). Some variation in film thickness is inherent in the VAC method, especially in cross-section films subjected to FIB and *a*-C sputtering. Energy dispersive X-ray spectroscopy (EDS) maps were collected in STEM mode to examine the penetration of the UiO-66-I layer into the mAu (Figure 3b). The strong Mα peak of Au is close to the Lα peak of Zr at ∼2 keV, so we monitored Zr at the Kα peak at 15.7 keV. The Zr signal appears deep inside the pores of the mAu, indicating that the UiO-66-I grows inside the porous film in the VAC method as well as forming the top surface of the film. The oxygen map appears to be strongest in the UiO-66-I film due to the ZrO_6_ nodes; there is also a faint signal from iodine. A more detailed STEM-EDS map of mAu@UiO-66-I and the substrate and SEM-EDS maps are shown in Figures S6 and S7. X-ray diffraction patterns of the mAu@UiO-66-I and UiO-66-I powder show the characteristic peaks of the UiO-66-I, indicating that the film is organized into the MOF framework (Figure S8a). The mAu@UiO-66-I and mAu films were also measured in X-ray photoelectron spectroscopy (XPS) and the survey spectra show that both the zirconium 3d peaks and iodine 3d peaks are only present in the mAu@UiO-66-I sample (Figure S8b). The surface energy of the Au surface and intermolecular forces between UiO-66-I precursors and Au will influence nucleation as well as diffusion and growth of the MOF films in VAC synthesis. Various MOFs have been synthesized inside more complex templates^42^ and on Au/Ag nanoparticle surfaces,^16,43^ thus these results show that infiltration of nanoscale patterns with MOFs is a tractable problem solved by experimentation.

**Figure 3:**
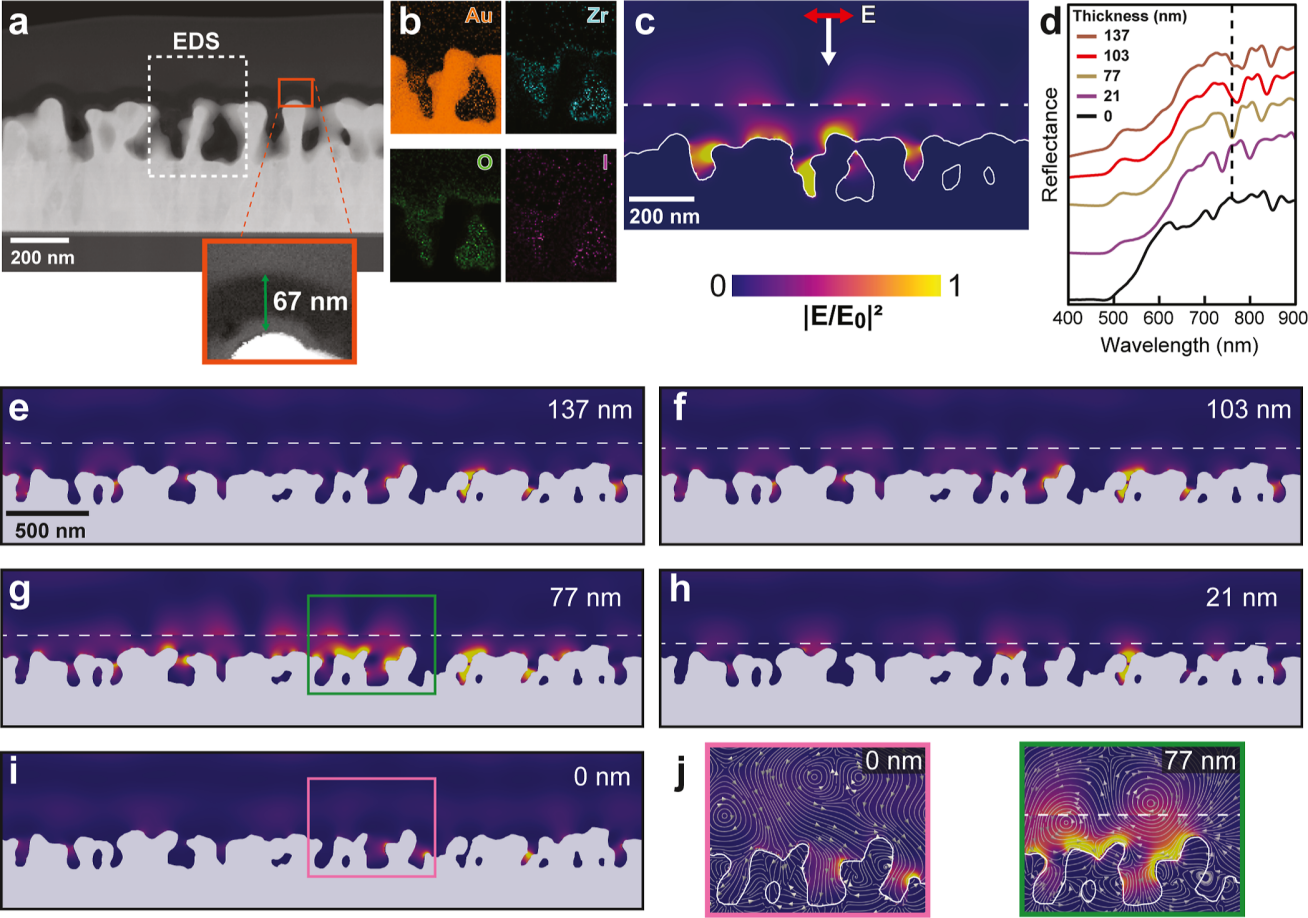
(a) HAADF-STEM image of a mAu@UiO-66-I cross-section that was created using FIB (see Figure S5). The inset image shows the UiO-66-I layer. (b) EDS maps measured on part of the cross-section in (a) showing the location of Au, Zr, O and I. (c) EM simulations using the cross-section in (a) with the optical constants for Au and UiO-66-I.^41,44^ The dotted line indicates the location of the top surface of UiO-66-I in the simulation. (d) EM simulations of the reflectance spectrum of mAu and mAu@UiO-66-I with different MOF film thicknesses (*t*_MOF_ = 0, 21, 77, 103, and 137 nm). (e–i) A larger image of the FIB cross-section of the mAu@UiO-66-I sample was used in these calculations and *E* ^2^ was monitored at λ = 759 nm to visualize how EM hotspots formed with the different UiO-66-I thicknesses *t*_MOF_ = 0, 21, 77, 103, and 137 nm. *E* ^2^ appears to be strongest at the mAu/MOF interface when the monitor wavelength overlaps the dips in the reflectance spectrum. (j) The zoomed-in *E* ^2^ maps of t_MOF_ = 0 and 77 nm show how the MOF generates more intense EM hotspots. Streamlines were overlaid on the *E* ^2^ maps to provide visual evidence of the EM energy flow and localization caused by the presence and absence of the MOF layer.

The CAH molecules need to traverse the thickness of the film to reach the EM hotspots near the mAu surface to be detected in SERS. To understand how UiO-66-I film thickness affects the distribution of EM hotspots, we loaded the full STEM image used to make Figure 3a into an EM solver, excited it with a plane wave, and applied optical constants for Au^44^ and UiO-66-I (*n* = 1.468).^41^ Figure 3c shows the EM intensity (*E* ^2^) map at λ = 785 nm with numerous hotspots on the top surface of the mAu and extending into the UiO-66-I film. The influence of junctions can be seen in the current density (*J*) plot (Figure S9), where large *J* is associated with the flow of electric charge caused by the excitation of SPs. EM hotspots tend to accumulate in the concave and convex regions adjacent to regions with higher *J*. Using the UiO-66-I film thicknesses determined by SEM (Figure S3a) we simulated the mAu film with *t*_MOF_ spanning 0–137 nm and plotted their reflection spectra in Figure 3d. The local disorder created by the mAu surface provides a broad distribution of momentum *k*-vectors where each site can couple to multiple different wavelengths^45^ based on its local environment and the incident angle of excitation, even in mAu films with no UiO-66-I layer. Increasing from *t*_MOF_ = 21–137 nm appears to redshift and strengthen the dips in the reflection spectrum, indicating that they correspond to different SP modes. Figure 3e–i shows the *E* ^2^ maps of the mAu@UiO-66-I cross sections at the different thicknesses while monitoring at λ = 759 nm to specifically observe the strongest resonance in the simulated mAu film with *t*_MOF_ = 77 nm. In the real films, the EM hotspots create a continuum of resonances spanning 500–900 nm wavelengths because of the multitude of local sites with morphologies that can couple to different wavelengths (Figure 2a).^45^ As the thickness of the MOF film increases, EM hotspots appear to increase in number and intensity and propagate into the dielectric layer, ostensibly as multiple scattering events generate TIR waves. Figure 3j shows the magnified sections of the *E* ^2^ maps of mAu with *t*_MOF_ = 0 and 77 nm. The time-averaged flow of energy is visualized here by converting the Poynting vector map into evenly spaced streamlines in ParaView. When the MOF film is 77 nm thick, compact clockwise and counterclockwise rotating vortices form near the mAu surface. The vortical cells concentrate and lift the EM field into the MOF, placing an EM hotspot near the adsorbed molecules, thereby boosting the SERS signal. MOF films thicker than *t*_MOF_ = 77 nm may also generate strong EM hot spots at the excitation wavelength, but 77 nm may be the ideal thickness that balances *E* ^2^ with the ability of the CAH to traverse the film and reach an EM hotspot. Also, multiple scattering effects in disordered but short-range correlated structures also make coupling to the plasmon resonance less angle-dependent.^46^ We simulated mAu@UiO-66-I film with *t*_MOF_ = 77 nm at incidence θ = 0–15° and still observed strong coupling (Figure S10). A substrate with less angle dependence could reduce some requirements and costs for the laser source and associated optics in SERS setups.

The SERS spectra generated with mAu@UiO-66-I were initially evaluated using 10^–6^ M DCB (Figure 4). All SERS measurements used mAu coated with the *t*_MOF_ = 77 nm films because it exhibited the strongest performance in initial SERS experiments with DCB (Figure S3) and calculations show sufficiently strong enhancement of EM hotspots within the MOF layer. The impact of MOF thickness on SERS performance stems from two factors:the UiO-66-I film’s ability to transport CAHs into EM hotspots, and the ability of the film to enhance coupling with incoming light. UiO-66-I supports numerous Raman vibrations in the 400–900 and 1200–1800 cm^–1^ regions (Figure 4a); thus a clean window with no MOF vibrations is ideal when using a MOF as the selectivity-imposing layer. However, chemometric methods and machine learning techniques offer powerful capabilities of suppressing background and interference signals that may enhance the detection of the analyte signals even inside MOFs.^47^ These methods were not necessary as DCB has a strong C–H ring breathing mode at 1001 cm^–1^ and an in-plane C–H bending vibration at 1084 cm^–1^ that gave an acceptable level of signal versus the background (see Tables S2 and S3 for detailed Raman assignments of spectra in Figure 4a). DCB interaction with mAu@UiO-66-I also generated a few new peaks that cannot be assigned to UiO-66-I or neat DCB which are indicated in Figure 4a using light blue circles. The peaks at 289 and 305 cm^–1^ could be ascribed to HaBs because HaB stretching modes tend to appear at low frequencies.^26^ The I 3d core-level XPS spectra collected from mAu@UiO-66-I films show a slight downshift and broadening after exposure to DCB, which is consistent with elevated electron density on the iodine atom as it serves as the σ-hole donor (Figure S11).^27,48,49^

**Figure 4:**
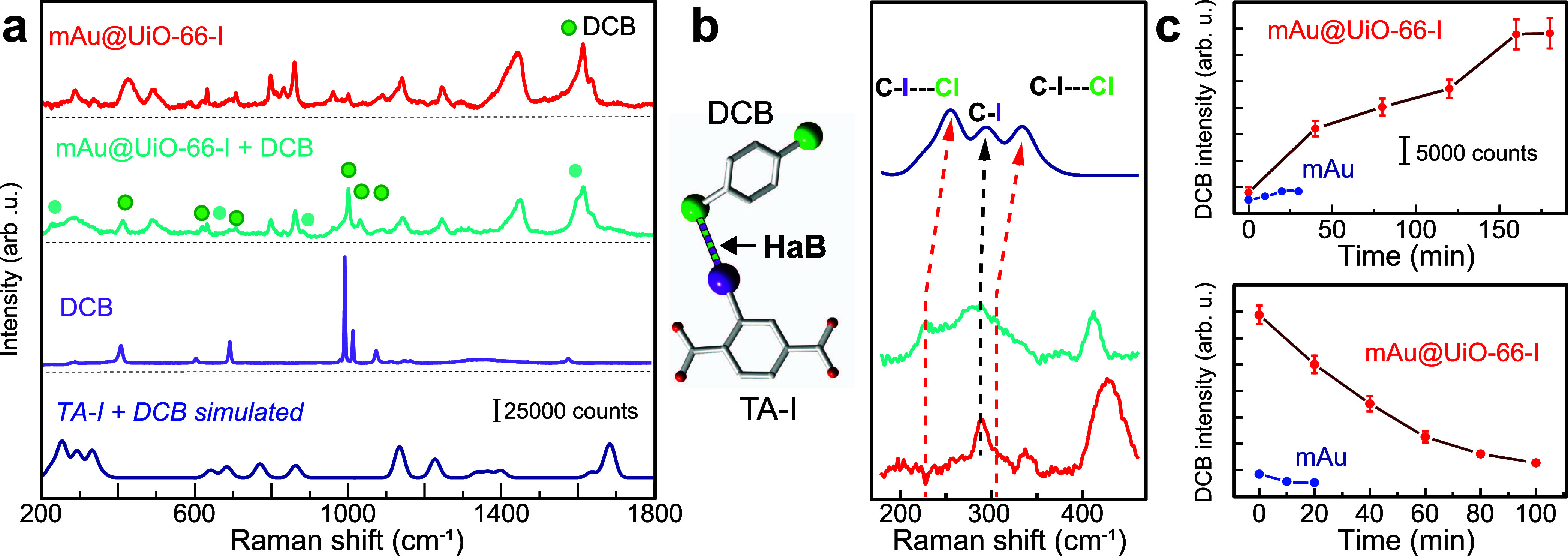
(a) Experimental SERS spectra of mAu@UiO-66-I, mAu@UiO-66-I with DCB and neat DCB. (b) DFT was used to calculate the Raman spectrum of 2-iodoterephthalic acid (TA-I) bonded to DCB via a halogen bond (HaB) in the low-frequency range. The experimental SERS spectra of mAu@UiO-66-I with DCB and mAu@UiO-66-I are plotted below in light blue and red, respectively. (c) Kinetic curves showing mAu@UiO-66-I (red points) and mAu (blue points) films being loaded with DCB (top graph) and then suspended in ethanol to remove the DCB (bottom graph). All SERS measurements used 785 nm laser excitation.

Density functional theory (DFT) calculations were performed on a model structure using 2-iodoterephthalic acid bonded to DCB via C–I---Cl HaB (Figure S12). The DFT computation shows that the C–I vibration is located at 300 cm^–1^, roughly matching the experimental peak for mAu@UiO-66-I at 289 cm^–1^ (Figure 4b). This peak broadens in the mAu@UiO-66-I films exposed to DCB, with an additional shoulder at 235 cm^–1^ that is ascribed to the C–I---Cl HaB bond. The peak at 305 cm^–1^ in the DFT computation could be an antisymmetric mode of the C–I---Cl HaB and the broadness of these peaks is likely due to the relatively weak (∼2 eV) nature of HaBs. The additional unidentified peaks at high wavenumbers in the full spectra are attributed to vibrational modes within the molecule that are affected by the presence of the HaB. Kinetic curves of DCB adsorption/desorption using the C–H 1001 cm^–1^ Raman peak were measured on mAu and mAu@UiO-66-I films exposed to 10^–6^ M DCB (Figure 4c; see raw data in Figure S13). The mAu@UiO-66-I shows rapid adsorption of DCB in the first 150 min despite the relative weakness of the HaB, consistent with the view that HaB interactions are quite stable even in aqueous solvent.^50^ DCB adsorption on mAu substrates equilibrated in 30 min and SERS intensity was ∼10× lower than mAu@UiO-66-I. Desorption of DCB from both mAu and mA@UiO-66-I films was initiated by placing the samples in a neat solution of ethanol, demonstrating the process is reversible. The mAu@UiO-66-I film was subjected to an additional 4 DCB absorption/desorption cycles with minimum loss in SERS performance (Figure S14). The SERS signal uniformity of the films was examined with a confocal Raman map (81 points; 5 μm pitch) of the mA@UiO-66-I films exposed to 0.5 mM DCB. The map gave a point-to-point relative standard deviation (RSD) of 13.4% for the 1001 cm^–1^ DCB band. For sample-to-sample tests, three droplets were analyzed similarly and yielded an RSD value of 15.2%. For substrate-to-substrate tests, three independently fabricated films were evaluated, yielding an RSD value of 17.8%. For comparison, “well-performing” SERS substrates based on thiol-terminated clean Au typically target RSD ≤ 15%.^51^ Achieving similar uniformity while imposing chemical selectivity through the MOF coating underscores the practical advantage of our system.

The US Environmental Protection Agency (EPA) set 5 × 10^–7^ M as the health-based guidance value for DCB in drinking water.^40^ To assess the limit of detection (LOD) of these substrates, we exposed the mAu@UiO-66-I films separately to DCB and 4-chlorobiphenyl (BiCl) solutions spanning 10^–4^ M to 10^–10^ M (Figures S15 and S16; and Tables S3 and S4 for peak assignment). The linear fit shows an excellent relationship with a correlation coefficient of >0.98 for both analytes with LOD of <10^–10^ M, which is well within the DCB levels set by the EPA. However, CAHs in the environment are more difficult to detect because environmental samples contain numerous organic and inorganic interference agents that could confuse SERS detection. Therefore, the role of the UiO-66-I film in this context is critical in imposing selectivity. Selectivity was initially examined using rhodamine 6G (R6G) on both mAu and mAu@UiO-66-I films in the presence of solutions of bovine serum albumin (BSA) protein, reference groundwater sample (GW; ERM-CA616) and marine water (MW) containing numerous different organic and inorganic materials found in nature (Figures S17 and S18; and SERS peak assignments for R6G and the various matrices in Tables S5–S10). The mAu films generate minimal SERS intensity when exposed to R6G, likely due to fouling of the Au surface, which restricts R6G access to the EM hotspots (see Note S2 for detailed discussion).^52^ In contrast, SERS measurements with mAu@UiO-66-I had no matrix-related peaks, indicating that most of the interfering agents were blocked.

Chemical selectivity as well as size discrimination in complex environments are some key elements that reticular chemistry can address via the selection of MOF metal atoms and linkers. The UiO-66-I must be capable of recruiting DCB from the environment and eschewing other contaminants while also enhancing EM hotspots in locations that are accessible to the DCB molecules (Figure 5a). Figure 5b,c shows SERS spectra of mixtures of DCB/BSA and DCB/GW measured with mAu and mAu@UiO-66-I films. BSA, GW and DCB are detectable on the mAu films due to a lack of selectivity. The mAu@UiO-66-I films omit any signal from the DCB and BSA. Moreover, the SERS signal of the DCB is strongly enhanced at the 1001 cm^–1^ C–H peak of DCB as expected due to a combination of selective recruitment via the HaB and enhanced EM hotspots for Raman excitation of DCB within the MOF layer. Finally, we examined the selectivity of the HaB by measuring mixtures of DCB, MW and naphthalene (Napht; peak assignment in Table S11) on mAu, mAu@UiO-66 and mAu@UiO-66-I films (Figure 5d,e). The mAu surface shows clear naphthalene peaks due to strong coupling of Au surface with the flat conjugated molecule. Both DCB and MW can be observed on mAu because SERS enhancement is unmediated. The mAu@UiO-66 films have no HaBs; thus MW, Napht and DCB appear in roughly similar intensities because the UiO-66 cannot form HaBs (Figure 5e). In turn, DCB generates the highest signal on mAu@UiO-66-I due to the HaBs. UiO-66-I selectively binds to CAHs over other PAHs and inorganic ions.

**Figure 5:**
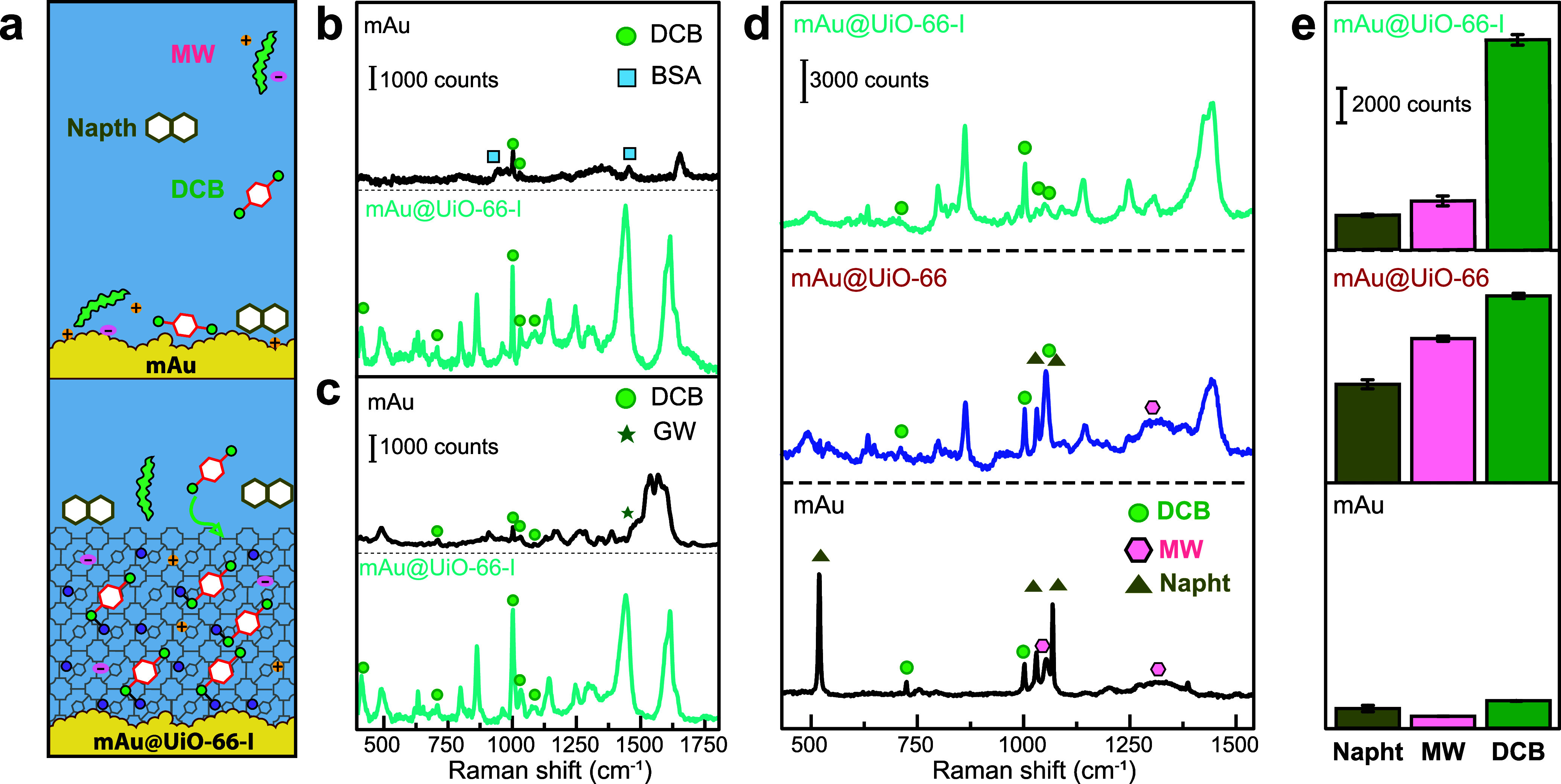
(a) An illustration showing how UiO-66-I mediates the interaction of different molecules and matrices with the plasmonically active mAu surface. SERS spectra of mAu and mAu@UiO-66-I films that were exposed to BSA (b) and GW (c), which were mixed with aqueous DCB (10^–6^ M). (d) mAu@UiO-66-I, mAu@UiO-66 and mAu films were exposed to mixtures of DCB, MW and Napht, and then the SERS spectra were collected. (e) A bar chart summarizing the SERS intensities for DCB, MW and Napht in (d).

## Conclusion

Hybridizing hyperuniform mAu films with UiO-66-I MOF coatings creates substrates that combine SERS enhancement with molecular selectivity. The hyperuniform disorder of the mAu films interacts with light to generate SP modes, which propagate and concentrate into broadly distributed EM hotspots as demonstrated by the Weibull analysis. In the context of SERS and EM enhancement, hyperuniform mesoporous Au films occupy a “Goldilocks zone” between crystalline order and random disorder—they retain just enough irregularity to generate intense EM hotspots, yet their long-range uniformity spreads those hotspots evenly across the surface. The MOF layer serves a dual function: it enhances light coupling into the mAu films and provides an iodine-functionalized framework that selectively recruits CAHs from the environment into the EM hotspots for SERS detection. Electromagnetic simulations reveal that the MOF layer enhances the confinement of SP modes and shifts their propagation closer to the top surface of the mAu film, improving accessibility to CAHs and amplifying the SERS signal. This synergy of chemical adsorption and photonic focusing concentrates both CAHs and light inside the MOF layer, driving detection limits below 10^–10^ M—several orders of magnitude lower than current environmental regulatory limits. The mAu@UiO-66-I substrates demonstrate robust selectivity in complex matrices, including mixtures containing proteins, PAHs, and reference samples from groundwater and marine water. Additionally, the relatively weak halogen bonding of adsorbed DCB allows for easy desorption, enabling the sensors to be reused multiple times.

Future research could explore MOFs with different chemistries to target other environmental contaminants or biomarkers. For instance, UiO-66-based MOFs with alternative functional groups might offer tunable selectivity and even stronger light coupling by leveraging higher refractive index materials (*n* > 1.78).^21,41^ The mAu films are type III hyperuniform materials, which is the weakest form of hyperuniformity.^31^ Further work on the self-assembly process may generate stronger forms of hyperuniformity where the interplay of long-range correlations and short-range disorder creates higher-performance SERS substrates at low cost.

## Methods

### Materials and Chemicals

Polystyrene-*block*–poly­(ethylene oxide) (PSS–PEO) block copolymer with a molecular weight of 18,000 and 7,500 for the polystyrene and poly­(ethylene oxide) blocks, respectively, was obtained from Polymer Source. Tetrahydrofuran (THF), dimethylformamide (DMF), absolute ethanol and bovine serum albumin (BSA) powder were purchased from Nacalai Tesque. Gold­(III) chloride trihydrate, zirconyl chloride, terephthalic acid, marine water reference (G0154) and groundwater reference (ERM-CA616) were obtained from Sigma-Aldrich. Additional materials used in the study include Fermfast ceramic Raschig rings (LD Carlson), Rhodamine 6G (TCI), 1,4-dichlorobenzene (AccuStandard), 4-chlorobiphenyl (Accustandard) and 2-iodoterephthalic acid (Ambeed).

### Preparation of Mesoporous Au (mAu)

Gold (Au) electrodes on silicon (Si) were prepared by sputtering 20 nm of Ti adhesion layer followed by 200 nm of Au on a Si wafer. The electrodeposition precursors were prepared as follows: 10 mg of PS_18,000_-*b*-PEO_7,500_ block copolymer was completely dissolved in 3 mL of THF at 40 °C, then 1.5 mL of ethanol, 1 mL of 40 mM aqueous HAuCl_4_, and 2.5 mL of Milli-Q deionized water (DIW; 18.2 MΩ·cm) were added dropwise in this sequence while stirring the solution. An electrochemical workstation (CH Instruments model 842BZ, USA) was used to deposit the mesoporous Au films on the Au electrodes using a conventional three-electrode system where Pt is the counter electrode, Ag/AgCl is the reference electrode, and the Au–Si substrate is the working electrode. At room temperature, the mAu film was deposited at −0.55 V (versus Ag/AgCl reference potential) for 3,000 s. Afterward, the substrates were immersed in THF at 40 °C to remove the PS_18,000_-*b*-PEO_7,500_ template and then the substrates were dried with N_2_ gas.^29^

### Preparation of Mesoporous Au Coated with UiO-66-I (mAu@UiO-66-I)

The MOF UiO-66 precursor solution was prepared by initially dissolving 7.5 mg of ZrOCl_2_·8H_2_O in 8 mL DMF and 26.4 μL acetic acid via ultrasonication, then mixing the solution with terephthalic acid (2.9 mM). This precursor solution was serially diluted with DMF to generate different MOF precursor solutions (2.9, 1.45, 0.96, 0.725, 0.483, 0 mM) to optimize the final thickness of the MOF film. The UiO-66-I precursor solutions were prepared using the same procedure but with a 50:50 terephthalic acid and 2-iodoterephthalic acid solution.^27^

The vapor-assisted conversion (VAC) method was used to coat mAu with UiO-66-I.^39^ VAC was performed in a 120 mL borosilicate bottle reactor (34 mm diameter) containing Raschig rings and an elevated sample stage (Figure S19). DMF (4.2 mL) and acetic acid (0.8 mL) were added to the Raschig rings, then a mAu substrate (5 mm × 5 mm) was placed on the sample stage and the mAu was coated with an 8.8 μL drop of the freshly prepared MOF precursor. The reactor was placed inside a preheated oven at 100 °C for 3 h. After the reaction, the substrate was removed from the reactor and placed in a vacuum oven at 80 °C for a minimum of 3 h before use in Raman measurements.

### Preparation of mAu@UiO-66-I Cross-Section

The mAu@UiO-66-I film was snapped in half with a diamond scribe and then coated with 30 nm of *a*-C. The sample was then placed in a dual FIB-SEM and additional *a*-C was deposited with the electron beam (5 kV) and ion beam (10 kV), followed by rough ion beam milling. The mAu@UiO-66-I sample was then fixed to a TEM grid using the lift-out technique and fine-milled to create the lamellar structure (Figure S5a).

### Pair Correlation Function and Window Variance Calculations

The locations of the pores in the SEM images were selected using ImageJ (Fiji). The image was denoised with a Gaussian algorithm (sigma = 1.0) using the DenoisEM plugin, inverted and the maxima were selected and assigned as pores. The pair correlation function *g*(*r*) was computed using different pore arrangements by first detecting pore centers using image processing techniques (thresholding and contour detection) and then converting their pixel coordinates to nanometer length scales. The function

$$g\left(r\right)=\frac{1}{2\pi r\rho N}\sum_{i\neq j}\delta \left(r-r_{ij}\right)$$

was implemented by using a KDTree data structure to efficiently calculate all pairwise distances *r*_*ij*_ between pore centers. The distances were then binned into a histogram with bin width d*r* = 2 nm to approximate the delta function δ. The histogram was normalized by the ideal gas expectation (2π*r*ρ d*r*) and the total number of particles *N* to obtain *g*(*r*), where ρ is the number density of pores calculated as *N* divided by the total area. This provides a measure of how the pore density varies as a function of distance compared to a random distribution, with *g*(*r*) = 1 indicating random ordering and peaks in *g*(*r*) revealing preferred separation distances in the pore arrangement.

Window variance analysis was performed to characterize the spatial distribution of pores within our images following the approach described by Torquato and Stillinger.^30^ The images were first converted to grayscale and then binarized using Otsu’s method,^53^ which selects an optimal global threshold by maximizing the between-class variance in pixel intensities. This thresholding allowed us to distinguish particles from the background effectively. We then randomly placed circular windows of radius *R* across the image and counted the number of particles *N*(*R*;*x*) within each window centered at position *x*. The average particle count for a given window *R* was calculated as

$$\left\langle N\left(R\right)\right\rangle =\frac{1}{M}\sum_{i=1}^{M}N\left(R;x_{i}\right)$$

where *M* is the total number of window placements. The variance of the particle counts was determined using

$$\sigma^{2}\left(R\right)=\frac{1}{M}\sum_{i=1}^{M}{\left(N\left(R;x_{i}\right)-\left\langle N\left(R\right)\right\rangle \right)}^{2}$$

By analyzing how the variance σ^2^(*R*) changes with the window radius *R*, we can infer the degree of uniformity or clustering in the 2D particle distribution. This approach allows a quantitative real-space assessment of spatial heterogeneity in the particle arrangements. The probability distribution and window variance calculations of the mAu, SQ and RNO patterns are shown in Figure S20 and Movie S1.

### Structural Characterization

SEM images were obtained using an FE-SEM Hitachi SU8320 (5 kV accelerating voltage) and FE-SEM Hitachi SU8000 + EDX with 20 kV accelerating voltage for EDX. STEM measurements were performed on a JEOL ARM200F equipped with a cold field emission gun operating at 200 kV with double aberration correctors and a resolution of 0.08 nm in STEM mode. Energy-dispersive X-ray spectroscopy (EDS) elemental maps were collected with a double silicon drift detector (detection surface area = 200 mm^2^; solid angle = 1.96 steradians). XPS was performed using an Omicron Nanotechnology ESCAProbeP spectrometer with a monochromated Al Kα X-ray source operating at 1,486.6 eV. The energy resolution was 50 eV for the initial survey and 2.5 eV for the high-resolution investigation. The analyzed area was 100 μm^2^. The concentrations of elements were calculated using atomic % (at. %) and the sensitivity factors provided by the manufacturer. XRD data was obtained using a Rigaku SmartLab X-ray diffractometer (Cu Kα radiation, 1.5406 Å) and a Bruker D2 Phaser second Generation diffractometer. Electrochemical active surface area (ECSA) measurements were performed in 0.5 M H_2_SO_4_ solution.

### EM Simulations

All simulations were performed using an EM solver (Lumerical). The mAu, SQ and RNO arrays were used as patterns to create 20 nm diameter nanoholes in a 3.0 μm × 0.6 μm flat gold film supercell using Au optical constants described by McPeak^44^ and periodic boundary conditions along the perimeter of the film. The nanohole arrays were excited with a plane wave polarized along the long axis of the films, and the electric field intensity (*E* ^2^) was monitored 3 nm above the top surface of the nanohole film. A 2× larger simulation was performed and the cumulative *E* ^2^ was within <5% of the original, indicating the supercell is sufficiently large to avoid spurious feedback. Slabs of UiO-66-I with different thicknesses were added to the top surface and holes mAu film by assigning the material a refractive index (*n*) of 1.468, which was extrapolated from ref. 41 to mimic the *n* of UiO-66-I at λ = 785 nm.

The cross-section lamellae from the STEM images were input into the simulations by taking the image and applying a threshold in Photoshop to remove all material except Au. This image was transformed into an STL file using Autodesk Fusion 360 and input into the EM solver. The lamella is ∼7.6 μm across and disordered, so we applied periodic boundary conditions along the length and width of the simulations and PML boundary conditions along the plane wave injection axis to simplify the calculations. The plane wave polarization was parallel to the long axis of the films. A power monitor was placed 1 μm above the lamella to measure the reflected light. Local fields were measured using a 3D field monitor. Slabs of UiO-66-I with *n* = 1.468 with different thicknesses were used to estimate the impact of MOF thickness on the reflectance and modal distribution in the Au pores and MOF films.

### Weibull Distribution Analysis

Electric-field intensity maps (|*E*|^2^/|*E*_0_|^2^) produced by identical EM simulations of hyperuniform (mAu), square (SQ) and random non-overlapping (RNO) gold nanohole arrays were first decorrelated by block-maxima declustering, where maps were partitioned into 57 nm × 57 nm blocks to match the measured nearest-neighbor distance in the mAu array (Figure 1b). Then the maximum value in each partition was used to ensure one datum per EM hotspot. These values were squared to obtain the theoretical SERS enhancement factor, EF = (|*E*|/|*E*_0_|)^4^. All EF values exceeding a threshold *U* = 2 were fitted by maximum likelihood to a two-parameter Weibull distribution, yielding the scale (*b*), which represents typical EF magnitude and shape (*c*). Larger *c* indicates greater uniformity. Parameter uncertainties (±1 σ) came from 2,000 bootstrap resamples of the exceedances, and a Kolmogorov–Smirnov test confirmed the fit (*p* is well above 0.05). Cross-sectional maps recorded normal to the film surface were analyzed identically but without declustering because the hotspots were already spatially isolated (Figure S4).

### Zeta Potential Measurements

Zeta-potential measurements were performed on an Otsuka Electronics ELSZ-2000ZS in a quartz cell measuring up to 120 V. Solutions were prepared as in the deposition solution and then diluted 1:3 using a solution with the same ratio of THF, ethanol and water to achieve an optimal intensity level on the detector. BCM solutions containing no HAuCl_4_ were prepared the same way but replacing the HAuCl_4_ solution with DI water.

### Absolute Reflectance Measurements

Absolute reflectance measurements were performed on a JASCO V770 UV–vis spectrophotometer with a diffuse reflectance accessory (SLM-907). The instrument was baselined with a manufacturer-calibrated aluminum mirror reference sample to collect absolute measurements on the Au, mAu and mAu@UiO-66-I films.

### Raman Spectroscopy and SERS Measurements

SERS spectra were measured using a Renishaw InVia Raman microscope equipped with a 785 nm laser (50 mW). The 785 nm laser wavelength was specifically selected to align with the broad resonance region of the substrate (500–900 nm, Figure 2a), to reduce fluorescence interference from organic analytes, and to minimize analyte degradation. A grating with 1,200 lines per mm was employed to balance spectral resolution with adequate coverage of the relevant wavenumber range (250–1,800 cm^–1^). A 50× magnification lens with a numerical aperture (NA = 0.75) was utilized to optimize both the spatial resolution and signal collection efficiency, thus maximizing Raman signal intensity. The resulting laser spot diameter on the sample was approximately 1.28 μm.

The laser power was carefully determined at 50 mW by gradually increasing intensity to achieve the highest Raman signal without causing observable sample damage or significant fluorescence; higher power resulted in analyte degradation, evidenced by visible white spots, whereas lower power significantly decreased signal intensity. Each spectrum was recorded with an integration time of 2 s accumulated over 40 measurements, a procedure chosen to substantially enhance the signal-to-noise ratio (SNR) while balancing against potential analyte degradation and elevated background signals.

The limit of detection (LoD) is defined as the lowest concentration at which the signal-to-noise ratio is equal to 3, following standard IUPAC methodology.^54^ Raman intensities of the characteristic peaks were measured across 5 concentrations ranging from 10^–4^ M to 10^–10^ M. A linear calibration curve of intensity vs −log­(concentration) was obtained (*R* ^2^ > 0.98). The LoD of our substrates is <10^–10^ M, approximately 5,000 times lower than the US EPA guidance level of 5 × 10^–7^ M.^40^ The reported LoD values were verified by averaging multiple (*N* = 10) independently measured spectra, each consisting of 40 accumulations (2 s each), to ensure repeatability and reliability.

Initially, 1,4-dichlorobenzene (DCB, 10^–6^ M) served as the model analyte to optimize the thickness of the UiO-66-I layer. Subsequently, dichlorobenzene and dichlorobiphenyl were used as target analytes. Rhodamine 6G (R6G, 10^–4^ M) was used for preliminary evaluations of filtering effects in complex matrices. Bovine serum albumin (BSA, 0.3 mg/mL), starch (0.3 mg/mL), naphthalene (10^–6^ M), groundwater ERM certified-CA616, and marine water (G0154) were employed to simulate environmental matrices encountered during SERS detection.

### Kinetic Studies on mAu@UiO-66-I

For SERS monitoring of HaB-related signals, mAu@UiO-66-I was immersed in 10^–6^ M solution of DCB and taken out for measurement after specific periods for spectra collection with experimental conditions explained above. After that, mAu@UiO-66-I was again immersed in DCB solution and the measurement procedure was repeated.

For SERS monitoring of HaB cleavage, mAu@UiO-66-I immersed in DCB for 200 min was transferred into ethanol and taken out for spectra collection. After that, mAu@UiO-66-I was again immersed in ethanol and the measurement procedure was repeated.

## Supplementary Material





## Notes

Structural characterization of short-range correlations; full Weibull statistical treatment of hotspot intensities; high resolution STEM, TEM and SEM images accompanied by STEM-EDS and SEM-EDS maps, XRD patterns of UiO-66-I films and powders; 3D EM simulations of the mAu@UiO-66-I cross-section and surface; DFT simulations of TA-I-DCB; XPS measurements; SERS measurements of DCB adsorption/desorption from the mAu@UiO-66-I and mAu films; sensing performance of mAu@UiO-66-I over 5 cycles; quantitative sensing of DCB with mAu@UiO-66-I; quantitative sensing of BiCl with mAu@UiO-66-I; SEM images of dried BSA, ERM-CA616 and Guillard’s (*F*/2) marine water enrichment solution (PDF)
Probability distributions and window variance calculations of the mAu, SQ and RNO patterns as the window radius changes from 0.05 to 0.6 microns (MP4)

## References

[1] Walker, C. H. Organochlorine insecticides. Organic Pollutants; CRC Press: Boca Raton, FL, 2008; pp 91–120.

[2] Yadav S., Kumar S., Haritash A. K. (2023). A Comprehensive
Review of Chlorophenols:
Fate, Toxicology and Its Treatment. J. Environ.
Manage..

[3] Qiao M., Cao W., Liu B., Zhao X., Qu J. (2017). Simultaneous Detection
of Chlorinated Polycyclic Aromatic Hydrocarbons with Polycyclic Aromatic
Hydrocarbons by Gas Chromatography–Mass Spectrometry. Anal. Bioanal. Chem..

[4] Schörnick C., Lüth A., Wobst B., Rotard W. (2021). Method Development
and Determination of Chlorinated Polycyclic Aromatic Hydrocarbons
in Different Matrices. Food Anal. Methods.

[5] Zhang D., Liang P., Ye J., Xia J., Zhou Y., Huang J., Ni D., Tang L., Jin S., Yu Z. (2019). Detection of Systemic Pesticide Residues in Tea Products
at Trace
Level Based on SERS and Verified by GC–MS. Anal. Bioanal. Chem..

[6] Carron K., Cox R. (2010). Qualitative Analysis and the Answer Box: A Perspective on Portable
Raman Spectroscopy. Anal. Chem..

[7] Losquin A., Camelio S., Rossouw D., Besbes M., Pailloux F., Babonneau D., Botton G. A., Greffet J. J., Stéphan O., Kociak M. (2013). Experimental Evidence of Nanometer-Scale Confinement
of Plasmonic Eigenmodes Responsible for Hot Spots in Random Metallic
Films. Phys. Rev. B.

[8] Langer J., Jimenez de Aberasturi D., Aizpurua J., Alvarez-Puebla R. A., Auguié B., Baumberg J. J., Bazan G. C., Bell S. E. J., Boisen A., Brolo A. G., Choo J., Cialla-May D., Deckert V., Fabris L., Faulds K., García
de Abajo F. J., Goodacre R., Graham D., Haes A. J., Haynes C. L. (2020). Present and Future of Surface-Enhanced Raman
Scattering. ACS Nano.

[9] Henzie J., Andrews S. C., Ling X. Y., Li Z., Yang P. (2013). Oriented Assembly
of Polyhedral Plasmonic Nanoparticle Clusters. Proc. Natl. Acad. Sci. U.S.A..

[10] La J. A., Lee H., Kim D., Ko H., Kang T. (2024). Enhanced Molecular
Interaction of 3D Plasmonic Nanoporous Gold Alloys by Electronic Modulation
for Sensitive Molecular Detection. Nano Lett..

[11] López-Puente V., Abalde-Cela S., Angelomé P. C., Alvarez-Puebla R. A., Liz-Marzán L. M. (2013). Plasmonic
Mesoporous Composites as Molecular Sieves
for SERS Detection. J. Phys. Chem. Lett..

[12] Leong S. X., Leong Y. X., Tan E. X., Sim H. Y. F., Koh C. S. L., Lee Y. H., Chong C., Ng L. S., Chen J. R. T., Pang D. W. C., Nguyen L. B. T., Boong S. K., Han X., Kao Y. C., Chua Y. H., Phan-Quang G. C., Phang I. Y., Lee H. K., Abdad M. Y., Tan N. S. (2022). Noninvasive and Point-of-Care Surface-Enhanced Raman Scattering (SERS)-Based
Breathalyzer for Mass Screening of Coronavirus Disease 2019 (COVID-19)
under 5 min. ACS Nano.

[13] Guselnikova O., Trelin A., Kang Y., Postnikov P., Kobashi M., Suzuki A., Shrestha L. K., Henzie J., Yamauchi Y. (2024). Pretreatment-Free SERS Sensing of Microplastics Using
a Self-Attention-Based Neural Network on Hierarchically Porous Ag
Foams. Nat. Commun..

[14] Kreno L. E., Greeneltch N. G., Farha O. K., Hupp J. T., Van Duyne R. P. (2014). SERS of
Molecules That Do Not Adsorb on Ag Surfaces: A Metal-Organic Framework-Based
Functionalization Strategy. Analyst.

[15] Guselnikova O., Lim H., Na J., Eguchi M., Kim H.-J., Elashnikov R., Postnikov P., Svorcik V., Semyonov O., Miliutina E., Lyutakov O., Yamauchi Y. (2021). Enantioselective SERS Sensing of
Pseudoephedrine in Blood Plasma Biomatrix by Hierarchical Mesoporous
Au Films Coated with a Homochiral MOF. Biosens.
Bioelectron..

[16] Wu Z. Y., Zhang M. M., Yang Y. Y., Han S., Li Y. T. (2023). Fabrication
of Core-Shell AuNP@UIO-66/Au Nanoparticles for in Situ SERS Monitoring
of the Degradation Process. New J. Chem..

[17] Chen H.-Y., Liu X.-B., Guan Q.-X., Zou C.-J., Fang P.-P. (2023). Detection
of Oxytetracycline on NH _2_ – UiO-66­(Zr)@Au NPs with
High Sensitivity and Selectivity by SERS. J.
Phys. Chem. C.

[18] Chen Z. C., Xu H. B., Chen H. Y., Zhu S. C., Huang W. F., He Y., Hafez M. E., Qian R. C., Li D. W. (2022). AuNPs-COFs Core-Shell
Reversible SERS Nanosensor for Monitoring Intracellular Redox Dynamics. Anal. Chem..

[19] Lai H., Li G., Xu F., Zhang Z. (2020). Metal-Organic Frameworks: Opportunities
and Challenges for Surface-Enhanced Raman Scattering – a Review. J. Mater. Chem. C.

[20] Yaghi O. M., O’Keeffe M., Ockwig N. W., Chae H. K., Eddaoudi M., Kim J. (2003). Reticular
Synthesis and the Design of New Materials. Nature.

[21] Treger M., Hannebauer A., Schaate A., Budde J. L., Behrens P., Schneider A. M. (2023). Tuning the Optical Properties of
the Metal–Organic
Framework UiO-66 *via* Ligand Functionalization. Phys. Chem. Chem. Phys..

[22] Lu G., Hupp J. T. (2010). Metal-Organic Frameworks
as Sensors: A ZIF-8 Based
Fabry-Pérot Device as a Selective Sensor for Chemical Vapors
and Gases. J. Am. Chem. Soc..

[23] Liu Y., Zhang Y., Tardivel M., Lequeux M., Chen X., Liu W., Huang J., Tian H., Liu Q., Huang G., Gillibert R., de la Chapelle M. L., Fu W. (2020). Evaluation of the Reliability
of Six Commercial SERS Substrates. Plasmonics.

[24] Vynck K., Pierrat R., Carminati R., Froufe-Pérez L. S., Scheffold F., Sapienza R., Vignolini S., Sáenz J. J. (2023). Light in
Correlated Disordered Media. Rev. Mod. Phys..

[25] De
Rosa C., Auriemma F., Diletto C., Di Girolamo R., Malafronte A., Morvillo P., Zito G., Rusciano G., Pesce G., Sasso A. (2015). Toward Hyperuniform Disordered Plasmonic
Nanostructures for Reproducible Surface-Enhanced Raman Spectroscopy. Phys. Chem. Chem. Phys..

[26] Cavallo G., Metrangolo P., Milani R., Pilati T., Priimagi A., Resnati G., Terraneo G. (2016). The Halogen Bond. Chem. Rev..

[27] Gulyaev R., Semyonov O., Mamontov G. V., Ivanov A. A., Ivanov D. M., Kim M., Švorčík V., Resnati G., Liao T., Sun Z., Yamauchi Y., Postnikov P. S., Guselnikova O. (2023). Weak Bonds,
Strong Effects: Enhancing the Separation Performance of UiO-66 toward
Chlorobenzenes via Halogen Bonding. ACS Mater.
Lett..

[28] Kalaj M., Momeni M. R., Bentz K. C., Barcus K. S., Palomba J. M., Paesani F., Cohen S. M. (2019). Halogen
Bonding in UiO-66 Frameworks
Promotes Superior Chemical Warfare Agent Simulant Degradation. Chem. Commun..

[29] Lim H., Kani K., Henzie J., Nagaura T., Nugraha A. S., Iqbal M., Ok Y. S., Hossain Md. S. A., Bando Y., Wu K. C. W., Kim H.-J., Rowan A. E., Na J., Yamauchi Y. (2020). A Universal Approach
for the Synthesis of Mesoporous
Gold, Palladium and Platinum Films for Applications in Electrocatalysis. Nat. Protoc..

[30] Torquato S., Stillinger F. H. (2003). Local Density
Fluctuations, Hyperuniformity, and Order
Metrics. Phys. Rev. E.

[31] Torquato S. (2018). Hyperuniform
States of Matter. Phys. Rep..

[32] Lee S. Y., Hung L., Lang G. S., Cornett J. E., Mayergoyz I. D., Rabin O. (2010). Dispersion in the SERS Enhancement
with Silver Nanocube Dimers. ACS Nano.

[33] Johnson P. B., Christy R. W. (1972). Optical Constants of the Noble Metals. Phys. Rev. B.

[34] Wen F., Zhang Y., Gottheim S., King N. S., Zhang Y., Nordlander P., Halas N. J. (2015). Charge Transfer Plasmons: Optical
Frequency Conductances and Tunable Infrared Resonances. ACS Nano.

[35] Nugraha A. S., Guselnikova O., Henzie J., Na J., Hossain M. S. A., Dag O ., Rowan A. E., Yamauchi Y. (2022). Symmetry-Breaking Plasmonic
Mesoporous Gold Nanoparticles with Large Pores. Chem. Mater..

[36] Ron R., Zielinski M. S., Salomon A. (2020). Cathodoluminescence Nanoscopy of
3D Plasmonic Networks. Nano Lett..

[37] Tavakoli N., Spalding R., Lambertz A., Koppejan P., Gkantzounis G., Wan C., Röhrich R., Kontoleta E., Koenderink A. F., Sapienza R., Florescu M., Alarcon-Llado E. (2022). Over 65% Sunlight
Absorption in a 1 μm Si Slab with Hyperuniform Texture. ACS Photonics.

[38] Galinski H., Favraud G., Dong H., Gongora J. S. T., Favaro G., Döbeli M., Spolenak R., Fratalocchi A., Capasso F. (2017). Scalable, Ultra-Resistant
Structural Colors Based on
Network Metamaterials. Light: Sci. Appl..

[39] Virmani E., Rotter J. M., Mähringer A., Von Zons T., Godt A., Bein T., Wuttke S., Medina D. D. (2018). On-Surface Synthesis
of Highly Oriented Thin Metal-Organic Framework Films through Vapor-Assisted
Conversion. J. Am. Chem. Soc..

[40] Office of Chemical Safety and Pollution Prevention . Para-Dichlorobenzene: Human Health Risk Assessment in Support of Registration Review (DP No. D438555); Office of Chemical Safety and Pollution Prevention: Washington, DC, 2018.

[41] Treger M., Hannebauer A., Behrens P., Schneider A. M. (2023). Development
of High Refractive Index UiO-66 Framework Derivatives *via* Ligand Halogenation. Phys. Chem. Chem. Phys..

[42] Li H., Qin Z., Yang X., Chen X., Li Y., Shen K. (2022). Growth Pattern
Control and Nanoarchitecture Engineering of Metal-Organic Framework
Single Crystals by Confined Space Synthesis. ACS Cent. Sci..

[43] Choi K. M., Kim D., Rungtaweevoranit B., Trickett C. A., Barmanbek J. T. D., Alshammari A. S., Yang P., Yaghi O. M. (2017). Plasmon-Enhanced
Photocatalytic CO_2_ Conversion within Metal-Organic Frameworks
under Visible Light. J. Am. Chem. Soc..

[44] McPeak K. M., Jayanti S. V., Kress S. J. P., Meyer S., Iotti S., Rossinelli A., Norris D. J. (2015). Plasmonic Films
Can Easily Be Better:
Rules and Recipes. ACS Photonics.

[45] Bosman M., Anstis G. R., Keast V. J., Clarke J. D., Cortie M. B. (2012). Light Splitting
in Nanoporous Gold and Silver. ACS Nano.

[46] Hwang V., Stephenson A. B., Magkiriadou S., Park J. G., Manoharan V. N. (2020). Effects
of Multiple Scattering on Angle-Independent Structural Color in Disordered
Colloidal Materials. Phys. Rev. E.

[47] Wang X., Sun X., Liu Z., Zhao Y., Wu G., Wang Y., Li Q., Yang C., Ban T., Liu Y., Huang J., Li Y. (2024). Surface-Enhanced Raman Scattering
Imaging Assisted by Machine Learning
Analysis: Unveiling Pesticide Molecule Permeation in Crop Tissues. Adv. Sci..

[48] Moreton J. C., Low J. X., Penticoff K. C., Cohen S. M., Benz L. (2022). An X-Ray Photoelectron
Spectroscopy Study of Postsynthetic Exchange in UiO-66. Langmuir.

[49] González L., Gimeno N., Tejedor R. M., Polo V., Ros M. B., Uriel S., Serrano J. L. (2013). Halogen-Bonding
Complexes Based on
Bis­(Iodoethynyl)­Benzene Units: A New Versatile Route to Supramolecular
Materials. Chem. Mater..

[50] Robertson C. C., Perutz R. N., Brammer L., Hunter C. A. A. (2014). Solvent-Resistant
Halogen Bond. Chem. Sci..

[51] Grys D., Chikkaraddy R., Kamp M., Scherman O. A., Baumberg J. J., de Nijs B. (2021). Eliminating Irreproducibility in SERS Substrates. J. Raman Spectrosc..

[52] Tezcan T., Boyaci I. H. (2021). A New and Facile
Route to Prepare Gold Nanoparticle
Clusters on Anodic Aluminium Oxide as a SERS Substrate. Talanta.

[53] Otsu N. (1979). A Threshold
Selection Method from Gray-Level Histograms. IEEE Trans. Syst., Man, Cybern..

[54] Thompson M., Ellison S. L. R., Wood R. (2002). Harmonized Guidelines
for Single-Laboratory
Validation of Methods of Analysis (IUPAC Technical Report). Pure Appl. Chem..

