# Supplementary material for: Hyperuniform Mesoporous Gold Films Coated with Halogen-Bonding Metal–Organic Frameworks for Selective Raman Sensing of Chlorinated Hydrocarbons
Source: ACS Nano. 2025 Jul 24;19(30):27890–901. doi: 10.1021/acsnano.5c09431 (PMC12333424; doi:10.1021/acsnano.5c09431)
Supplement: Supplementary file 1 [file nn5c09431_si_001.pdf]

Hyperuniform Mesoporous Gold Films Coated with Halogen-Bonding Metal-Organic Frameworks for Selective Raman Sensing of Chlorinated Hydrocarbons

Sarah Z. Khairunnisa[1,2,3], Olga Guselnikova[1], Yunqing Kang[4], Pavel S. Postnikov[5], Rashid R. Valiev[5], Jonathan P. Hill[1], Nugraha Nugraha[2,3], Brian Yuliarto[2,3], Yusuke Yamauchi[1,4,6,*], Joel Henzie[1,*]

[1] International Center for Materials Nanoarchitectonics (WPI-MANA), National Institute for Materials Science (NIMS), 1-1 Namiki, Tsukuba, Ibaraki 305-0044, Japan.

[2] Doctoral Program of Nanoscience and Nanotechnology, Graduate School, Institut Teknologi Bandung, Bandung 40132, Indonesia.

[3] Research Center for Nanoscience and Nanotechnology (RCNN), Institut Teknologi Bandung, Bandung 40132, Indonesia.

[4] Department of Materials Process Engineering, Graduate School of Engineering, Nagoya University, Nagoya 464-8603, Japan.

[5] Research School of Chemistry and Applied Biomedical Sciences, Tomsk Polytechnic University, 43A Lenin Avenue, Tomsk, 634050, Russian Federation.

[6] School of Chemical Engineering, Australian Institute for Bioengineering and Nanotechnology (AIBN), The University of Queensland, Brisbane, QLD 4072, Australia.

Email: y.yamauchi@uq.edu.au(Y.Y.); HENZIE.Joeladam@nims.go.jp (J.H.)

Supplemental Information

Table of Contents

Supplemental Note 1. A simple calculation describes the concentration of block copolymer micelles in the electrodeposition solution.

Supplemental Figure 1. Assignment of pores to a 3 × 3 μm top-surface SEM image of an mAu film. 2D patterns of pores with a square array and random non-overlapping distributions were created for comparison.

Supplemental Figure 2. Full Weibull fits of the mAu, SQ and RNO arrays shown in **Figure 1f**.

Supplemental Figure 3. The thickness of UiO-66-I grown on the mAu surface was modulated by changing MOF precursor concentration. SERS intensity of DCB at 1001 cm^{-1} was used to evaluate the impact of UiO-66-I film thickness on SERS intensity.

Supplemental Figure 4. Weibull fit and KS test on the thickness simulations on mAu@UiO-66-I.

Supplemental Figure 5. STEM images of the mAu@UiO-66-I FIB cross-section mounted on a TEM grid.

Supplemental Figure 6. Additional STEM-EDS maps of the mAu@UiO-66-I cross-section shown in **Figure 3a**.

Supplemental Figure 7. Top-side SEM-EDS maps of a mesoporous Au film coated with UiO-66-I MOF using a 1.45 mM precursor MOF solution.

Supplemental Figure 8. XRD patterns of mAu@UiO-66-I films versus UiO-66-I powder, and XPS survey spectra of mAu and mAu@UiO-66-I films.

Supplemental Figure 9. Current density (J) plots showing large J is associated with the flow of electric charge, causing EM hotspots to form in the adjacent regions.

Supplemental Figure 10. EM simulations of mAu@UiO-66-I using t = 77 nm and changing the angle of incidence of the plane wave (θ = 0° to 15°).

Supplemental Figure 11. High-resolution core-level 3*d* XPS spectra of a mAu@UiO-66-I film before and after being exposed to 1,4-dichlorobenzene (DCB).

Supplemental Figure 12. (**a**) The optimized geometry of iodine-functionalized terephthalic acid ligand (I-TA) with dichlorobenzene (DCB) and (**b**) comparison between the experimental Raman spectrum and the computed harmonic normal modes.

Supplemental Figure 13. SERS measurements of mAu@UiO-66-I (**a**) and mAu (**b**) after immersion into DCB solution for specific periods and after immersion of mAu@UiO-66-I (**c**) and mAu (**d**) with absorbed DCB into ethanol to cleave HaB for specific periods

Supplemental Figure 14. mAu@UiO-66-I performance of DCB sensing in multiple sensing cycles.

Supplemental Figure 15. (**a**) Quantitative SERS sensing of DCB using concentrations 10^{-4} to 10^{-10} M. (**b**) The corresponding characteristic DCB peak intensity at 1001 cm^{-1} and 710 cm^{-1} as a function of the concentration.

Supplemental Figure 16. (**a**) Quantitative SERS sensing of BiCl using concentrations 10^{-4} to 10^{-10} M. (**b**) The corresponding characteristic BiCl peak intensity at 1004 cm^{-1} as a function of the concentration.

Supplemental Figure 17. SEM images of a silicon wafer after applying a drop of the following solutions and drying them: (**a**) BSA, (**b**) ERM-CA616 groundwater reference and (**c**) Guillard's (F/2) marine water enrichment solution.

Supplemental Figure 18. SERS sensing of R6G (10^{-4} M) in the presence of interfering components of water: (**a**) common components are bovine serum albumin (BSA), groundwater (GW) and marine water (MW), were mixed with R6G before detection. (**b**) SERS spectra of R6G on mAu and mAu@UiO-66-I, mixed with (**c**) BSA, (**d**) GW and (**e**) MW. (**f**) The sensitivities of SERS detection with/without interfering components.

Supplemental Note 2. A further explanation of the samples and matrices in **Supplemental Figure 18**.

Supplemental Figure 19. An illustration of the VAC setup used to coat mAu films with UiO-66-I.

Supplemental Figure 20. Particle distribution maps (**top row**; square array, mesoporous Au, random non-overlapping distribution), probability densities (**middle row**), and window variance (**bottom row**) showing the final frame from **Supplemental Movie 1**.

Supplemental Table 1. Weibull parameters and KS fit parameters for the mAu, SQ and RNO simulated substrates.

Supplemental Table 2. Assignment of the Raman peaks in **Fig. 4a**: mAu@UiO-66-I.

Supplemental Table 3. Assignment of the Raman peaks in **Fig. 4a**: sensing of 1,4-dichlorobenzene (DCB) on mAu@UiO-66-I.

Supplemental Table 4. Assignment of the Raman peaks in **Supplemental Fig. 16**: sensing of 4-chlorobiphenyl (BiCl) on mAu@UiO-66-I.

Supplemental Table 5. Assignment of the Raman peaks in **Supplemental Fig. 18**: sensing of R6G on mAu@UiO-66-I.

Supplemental Table 6. Assignment of the Raman peaks of BSA powder (**Fig. 5b**; **Supp. Fig. 18**).

Supplemental Table 7. Composition of the certified groundwater reference material ERM-CA616.

Supplemental Table 8. Composition of the certified marine water reference material G0154-500ML (Sigma-Aldrich).

Supplemental Table 9. Assignment of the Raman peaks of groundwater dried on a silicon wafer (**Fig. 5c**; **Supp. Fig. 18**).

Supplemental Table 10. Assignment of the Raman peaks for marine water dried on a silicon wafer (**Fig. 5d,e**; **Supp. Fig. 18**).

Supplemental Table 11. Assignment of the Raman peaks of naphthalene (**Fig. 5d,e**)

Supplemental Note 1:

An example calculation using a 25 nm diameter block copolymer micelles (BCM) to assess the crowded molecular environment of the electrodeposition solution. We consider a block copolymer comprising a PSS segment (18,000 g/mol) and a PEO segment (7,500 g/mol) for a total chain molecular weight of 25,500 g/mol. The mass per chain is given by:

Mass per chain (m_chain**):**
m_chain = M_chain / N_A = (25,500 g/mol) / (6.022×10^{-23} mol^{-1}) ≈ 4.234×10^{-20} g/chain

Volume per chain (V_chain**):**
Assuming a density of ρ *=1.2 g/cm^3, the volume occupied by a single chain is:*

V_chain = m_chain / ρ = (4.234×10^{-20} g) / (1.2 g/cm^3) ≈ 3.528×10^{-20} cm^3/chain

Convert volume to nm^3 (1 cm^3 = 10^{21} nm^3):

V_chain ≈ 35.28 nm^3/chain

Volume of Micelle (V_micelle**):**
For a micelle with a 25 nm diameter (radius r = 12.5 nm), the micelle volume is:

V_micelle = $(4/3)\pi r^3 = (4/3)\pi(12.5\ nm)^3 \approx 8,180\ nm^3$

Aggregation Number (N_agg**):**
To determine how many block copolymers are required to form the micelle (aggregation number) we must divide the micelle volume by the chain volume.

N_agg = V_micelle / V_chain = 8,180 nm^3 / 35.28 nm^3 ≈ 232 chains/micelle

Number of micelles in the electrodeposition solution (N_micelles**):**
To create the electrodeposition solution we dissolve 10 mg of polymer (0.01 g) in 8 mL of solution containing THF, ethanol, gold precursor and water.

N_chains = (m_polymer) / (m_chain) = 0.01 g / (4.234×10^{-20} g) ≈ 2.36×10^{17} chains
N_micelles = N_chains / N_agg = (2.36×10^{17}) / 232 ≈ 1.018×10^{15} micelles

For a solution volume of 8 mL, (1 mL = 10^{12} μm^3):

V_solution = 8 mL × 10^{12} μm^3/mL* ≈ 8×10^{12} μm^3

Micelle Concentration (C_micelles**) in micelles/μm^3:**
C_micelles = N_micelles / V_solution = (1.0×10^{15}) / (8×10^{12}) ≈ 127 micelles/μm^3

Micelle Spacing assuming a uniform distribution in solution (d_micelle**):**
d_micelle ≈ $(1 / 127\ \mu m^{-3})^{1/3}$ ≈ 0.2 μm ≈ 200 nm

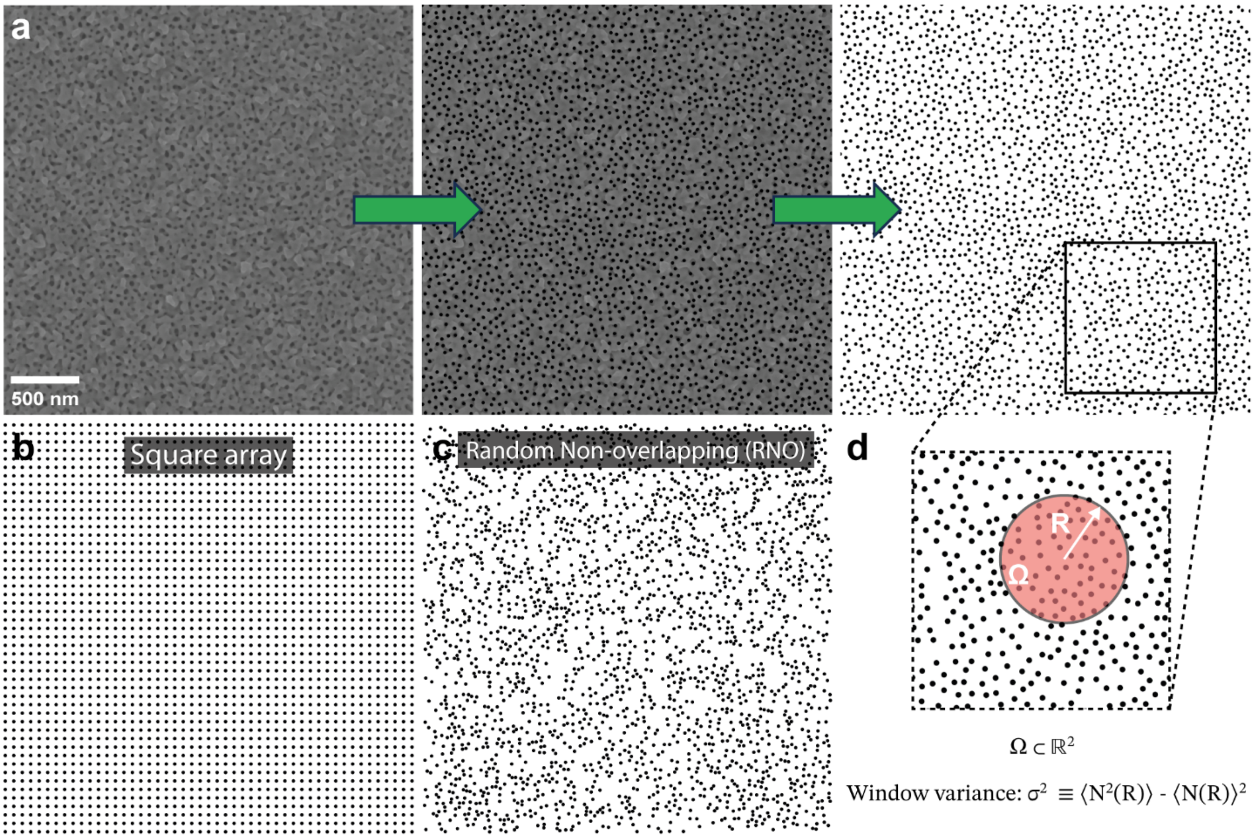

Supplemental Figure 1. (**a**) 3 × 3 μm SEM image of the top surface of mAu films was processed with ImageJ and 2385 pores were assigned based on contrast. A similar number of pores was distributed in a 3 × 3 μm box to create a periodic square array (**b**) and a random non-overlapping distribution (**c**). (**d**) Window variance calculation samples a region that is a subset of a 2D Euclidean space. A circular window of varying diameter is used to quantify the fluctuation of the number of points around its mean.

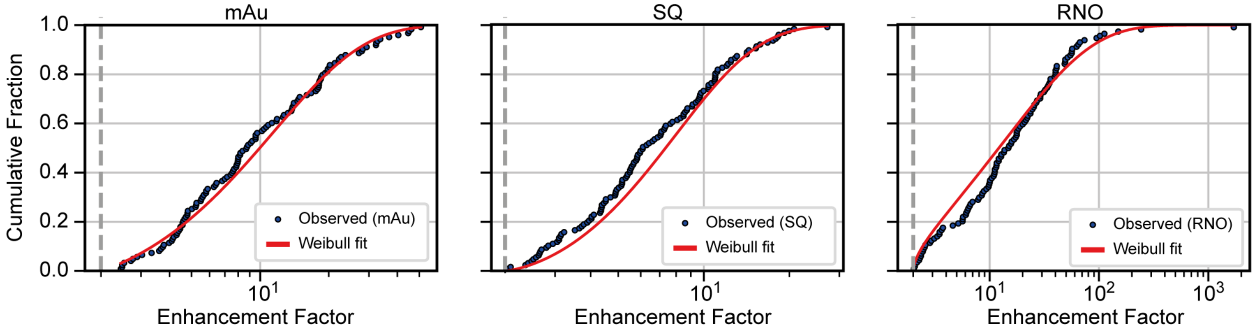

Supplemental Figure 2. Full Weibull fits of the mAu, SQ and RNO arrays shown in **Figure 1f**.

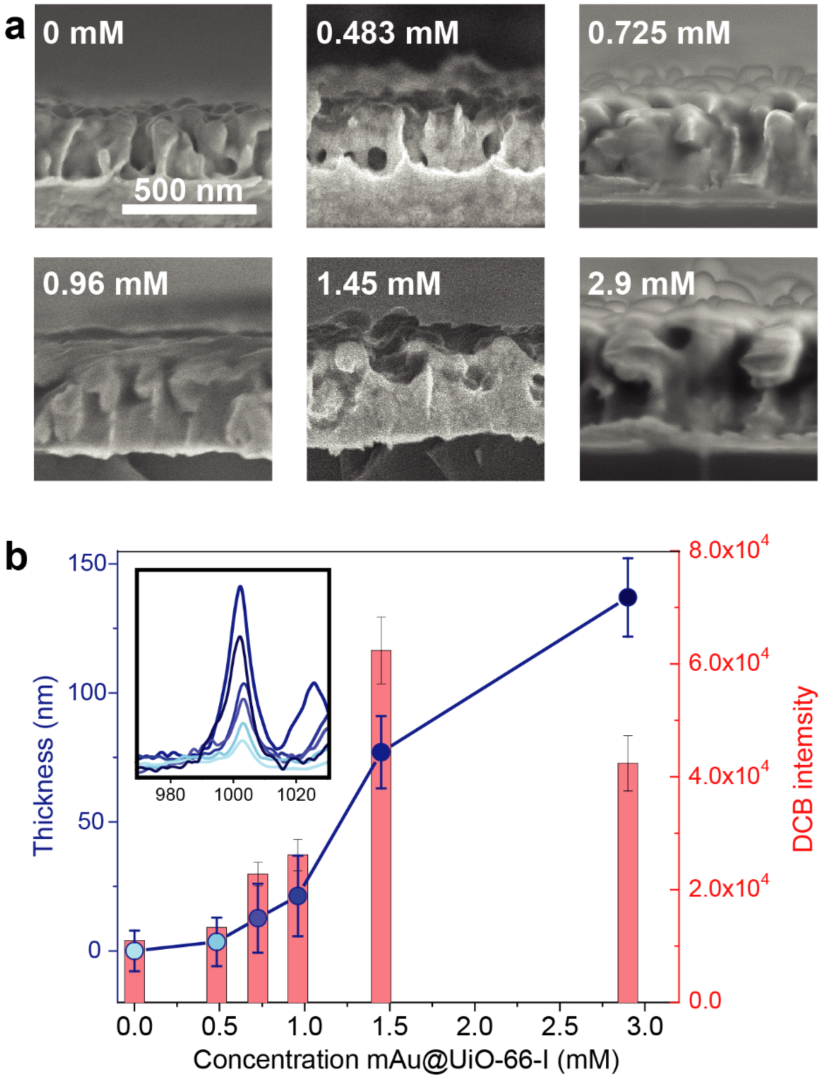

Supplemental Figure 3. The thickness of UiO-66-I grown on the mAu surface was modulated by changing MOF precursor concentration. (**a**) SEM images of mAu@UiO-66-I cross-sections from each precursor concentration. (**b**) A graph showing the thickness of the UiO-66-I film generated by each concentration (y-axis; left) and SERS intensity generated by soaking these samples in DCB (10^{-6} M) solution (y-axis; right). The inset in (**b**) shows the DCB peak at 1001 cm^{-1} used to assess SERS intensity.

a

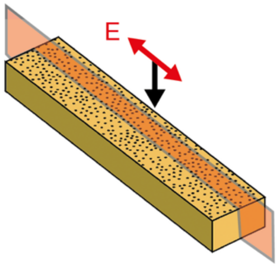

b

Sample	Scale Factor (*b*)	Shape Factor (*c*)	KS D	KS p
mAu	16.8 ± 3.77	0.588 ± 0.043	0.085	0.69
mAu-MOF21	13.8 ± 2.12	0.754 ± 0.054	0.047	0.987
mAuMOF77	20.2 ± 2.90	0.604 ± 0.029	0.067	0.457
mAuMOF103	19.3 ± 2.62	0.616 ± 0.029	0.063	0.536
mAuMOF137	18.2 ± 2.56	0.639 ± 0.032	0.064	0.575

Supplemental Figure 4. (**a**) Illustration showing how the E^2 monitor is oriented orthogonal to the mAu array surface as a cross-section to examine how increasing thicknesses of MOF with a refractive index of 1.468 affect frequency and strength of EM hotspots that contribute to SERS EF. (**b**) A table showing the Weibull fit parameters and Kolmogorov–Smirnov (KS) test parameters of the thickness experiment.

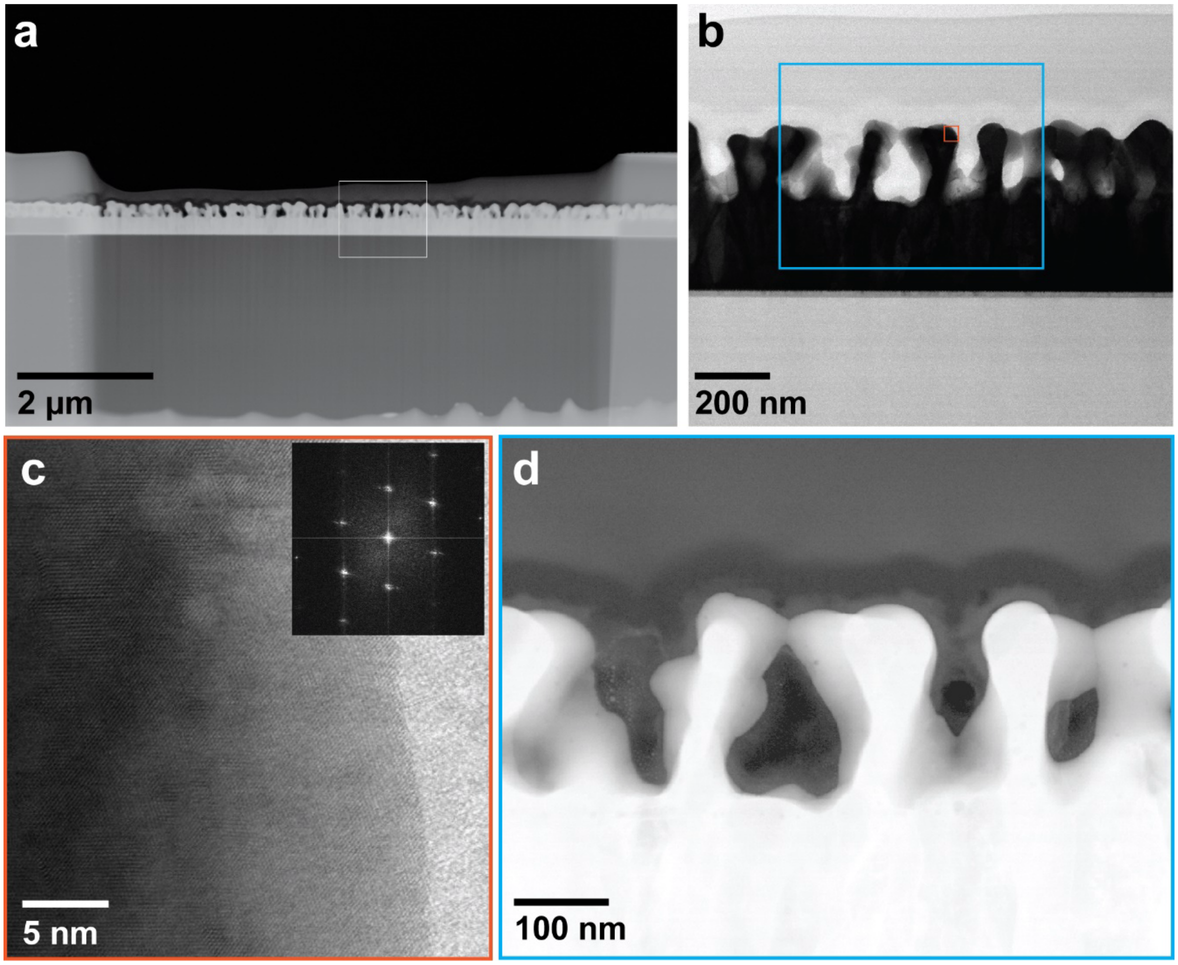

Supplemental Figure 5. (**a**) Dark-field STEM image showing the FIB cross-section of the mesoporous Au film coated with UiO-66-I on a TEM grid. A carbon film was deposited on the topside of the grid to fix the sample on the TEM grid. A zoom-in bright-field STEM image of the film in the white box is shown in (**b**). This area was analyzed carefully with STEM. The atomic structure of the mesoporous Au located in the red box is shown in (**c**). The inset Fourier transform of (**inset; c**) shows the characteristic diffraction pattern of Au metal. (**d**) The DF-STEM image of the film shows the morphology of the UiO-66-I film on the surface of the mAu and inside the pores. The MOF coats the interior of the pores conformally, but there are holes in the MOF due to some combination of imperfect coating, beam damage from the FIB, and beam damage from the 200 kV TEM.

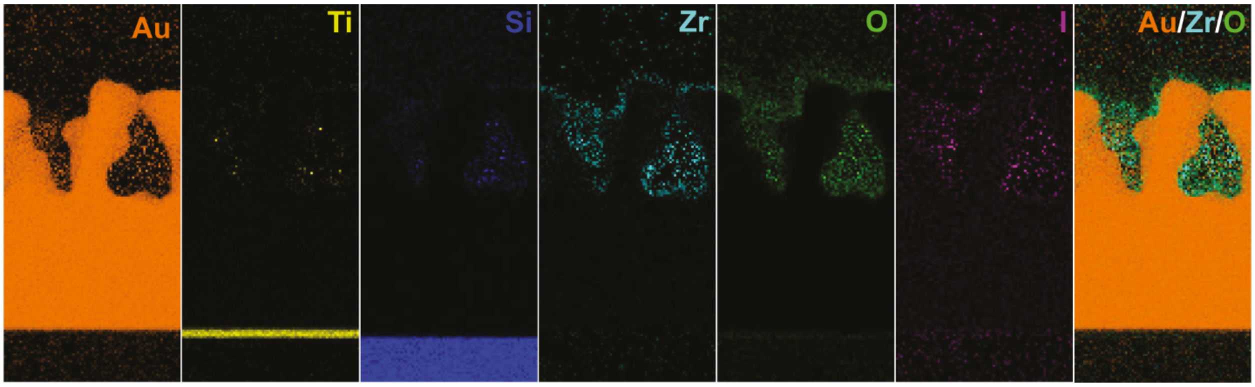

Supplemental Figure 6. Additional STEM-EDS maps of the mAu@UiO-66-I and substrate cross-section shown in **Figure 3a**.

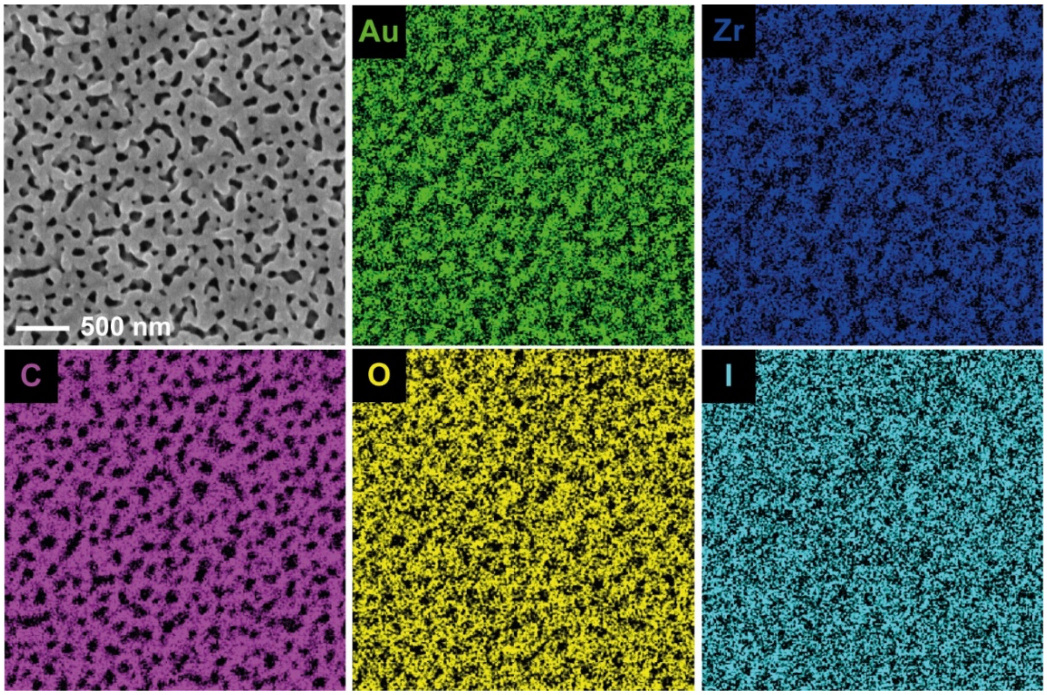

Supplemental Figure 7. Top-side SEM-EDS map of a mesoporous Au film coated with UiO-66-I MOF using a 1.45 mM precursor MOF solution. The EDS maps show locations in the EDS spectrum where Au, Zr, C, O and I absorb the electron beam and emit X-rays.

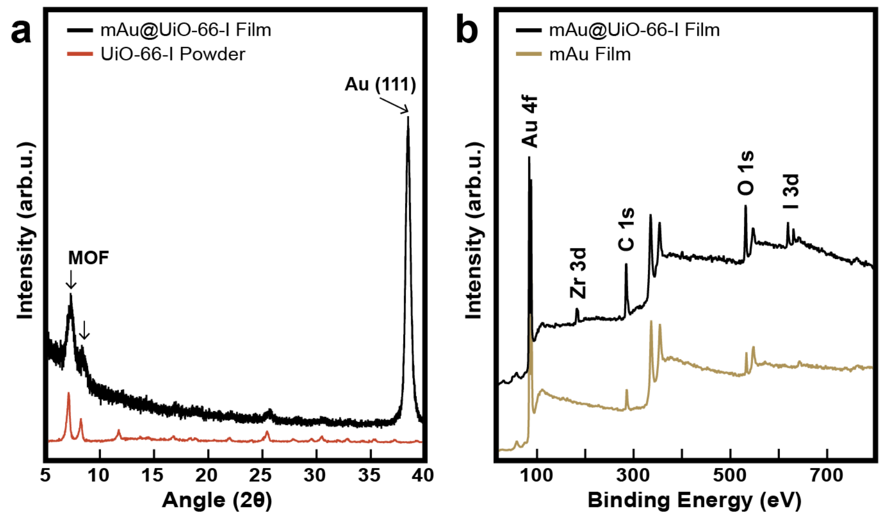

Supplemental Figure 8. Top (**a**) XRD patterns of the mAu@UiO-66-I film compared to a UiO-66-I powder sample. (**b**) XPS survey spectra of the mAu@UiO-66-I film versus a mAu film containing only Au.

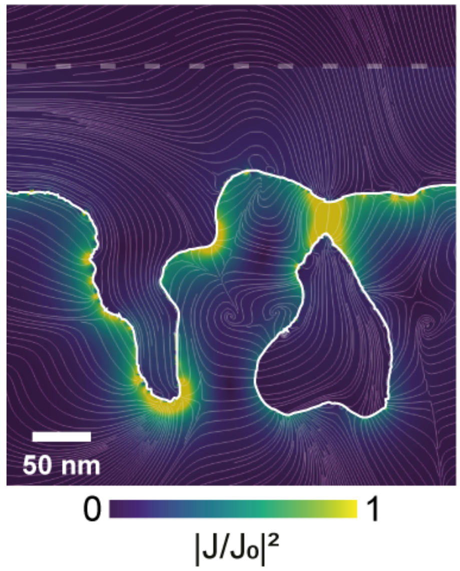

Supplemental Figure 9. Current density (J) plots showing large J is associated with the flow of electric charge, causing EM hotspots to form in the adjacent regions.

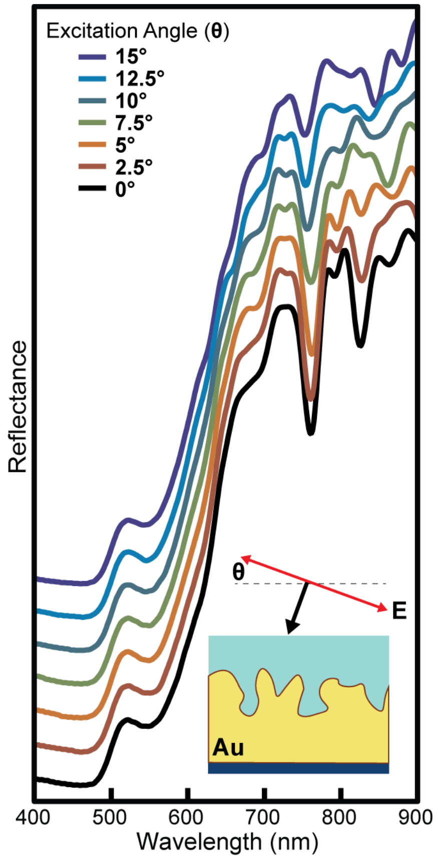

Supplemental Figure 10. EM simulations of mAu@UiO-66-I infiltrated with a 77 nm thick dielectric slab (n = 1.468) and changing the angle of incidence of the plane wave (θ = 0° to 15°).

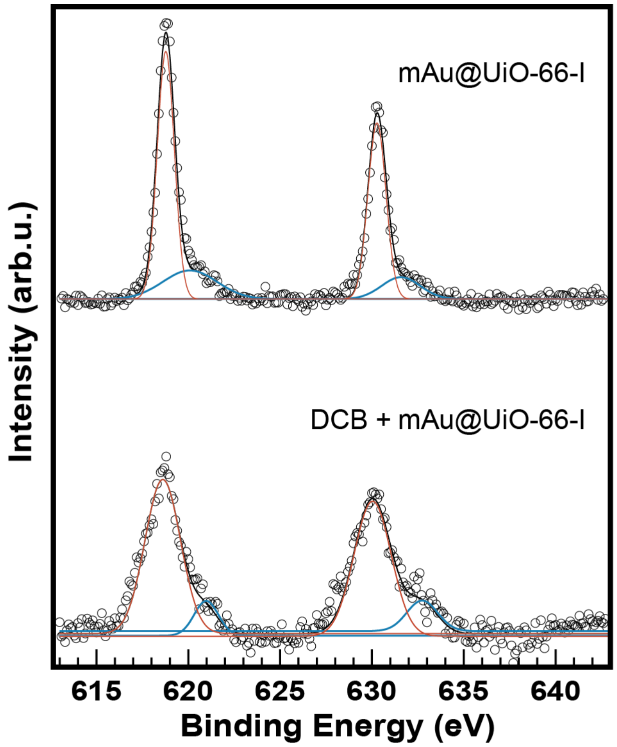

Supplemental Figure 11. High-resolution XPS of the I $3d_{5/2}$ and I $3d_{3/2}$ peaks of the mAu@UiO-66-I film and a mAu@UiO-66-I film exposed to 1,4-dichlorobenzene (DCB).

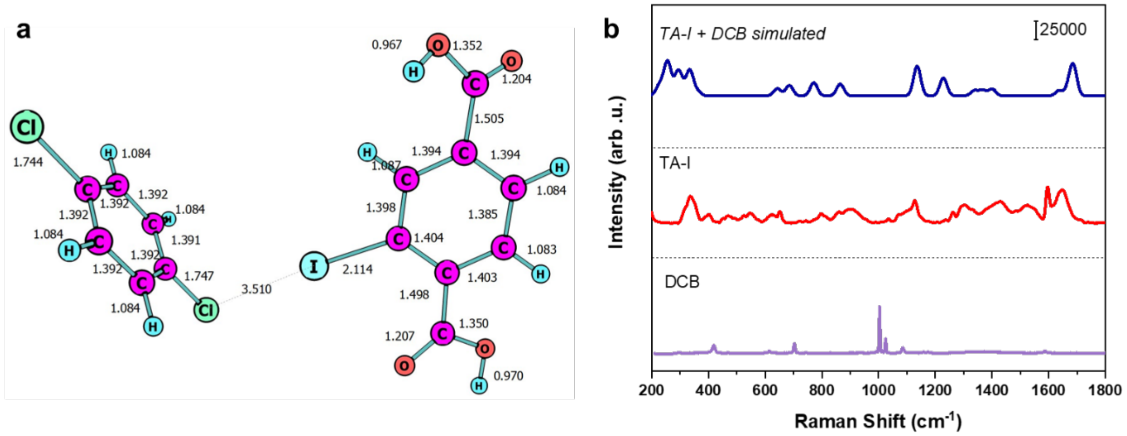

Supplemental Figure 12. (**a**) The optimized geometry of 2-iodoterephthalic acid ligand (I-TA) with 1,4 dichlorobenzene (DCB) and (**b**) comparison between the experimental Raman spectrum and the computed harmonic normal modes.

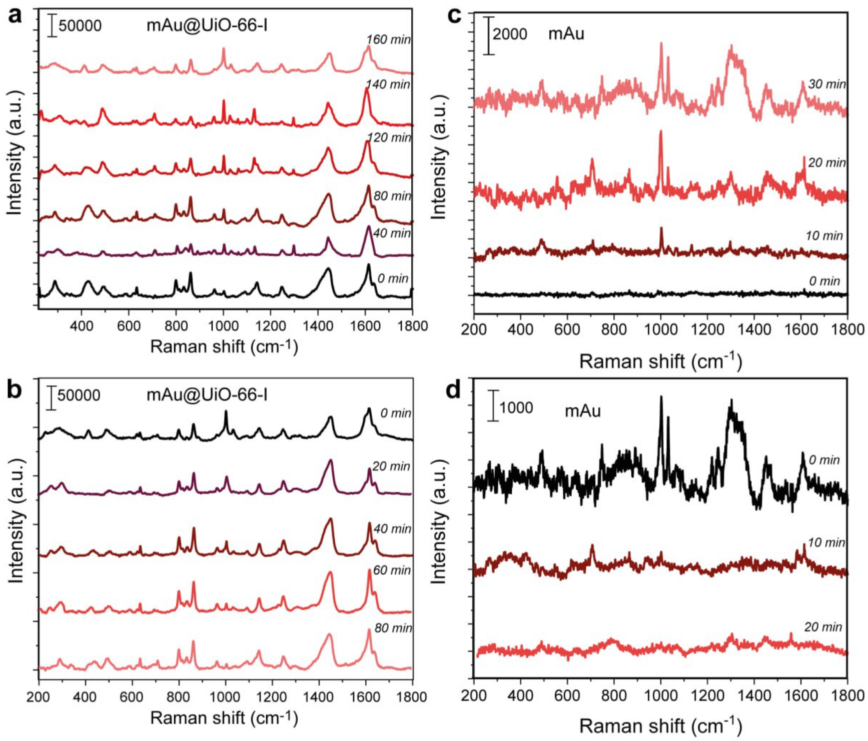

Supplemental Figure 13. (**a**) SERS measurements of mAu@UiO-66-I as it is immersed in DCB over time. (**b**) SERS measurements of mAu@UiO-66-I after it was immersed in neat ethanol to cleave the HaB over time. (**c**) SERS measurements of mAu as it is immersed in DCB over time. (**d**) SERS measurements of mAu after it was immersed in neat ethanol to cleave the HaB over time.

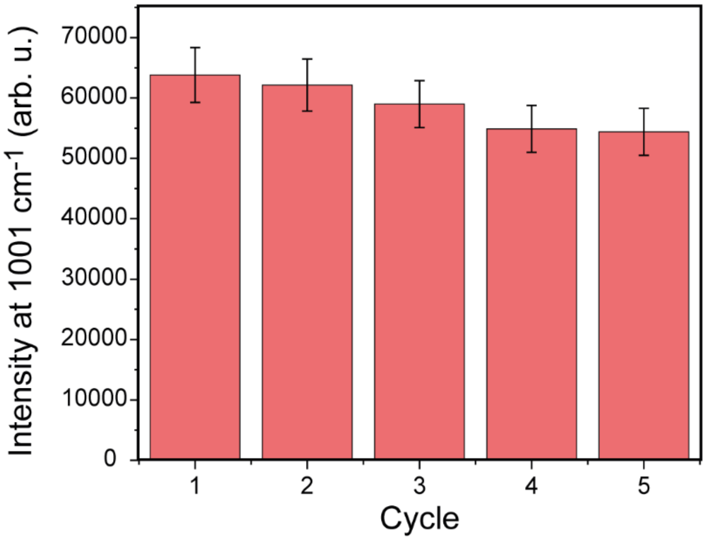

Supplemental Figure 14. mAu@UiO-66-I performance of DCB sensing in multiple sensing cycles.

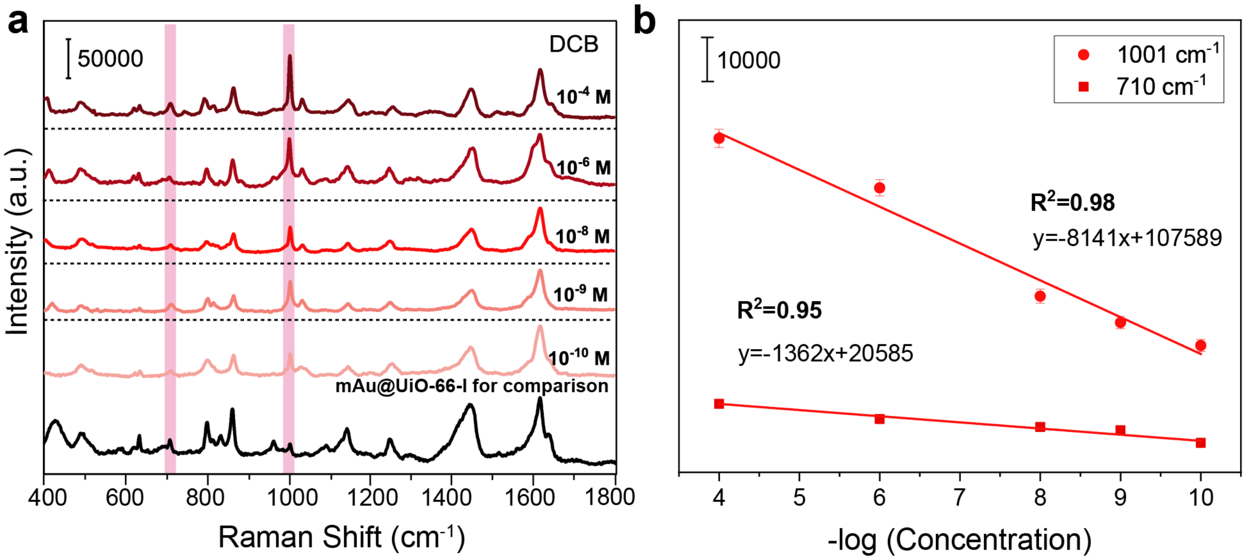

Supplemental Figure 15. (**a**) Quantitative SERS sensing of DCB using concentrations 10^{-4} to 10^{-10} M, (**b**) the corresponding characteristic DCB peak intensities at 1001 cm^{-1} and 710 cm^{-1} as a function of the concentration.

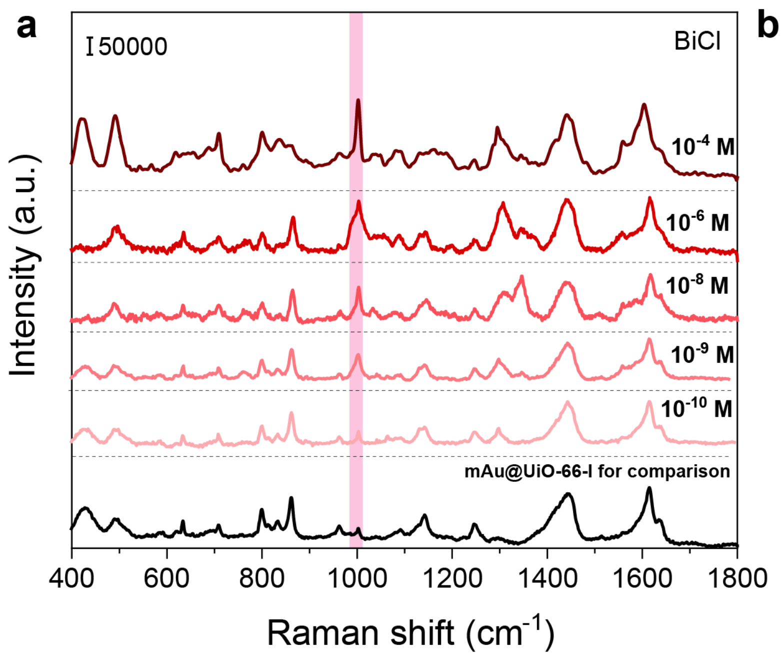

Supplemental Figure 16. (**a**) Quantitative SERS sensing of BiCl using concentrations 10^{-4} to 10^{-10} M. (**b**) The corresponding characteristic BiCl peak intensity at 1004 cm^{-1} as a function of the concentration.

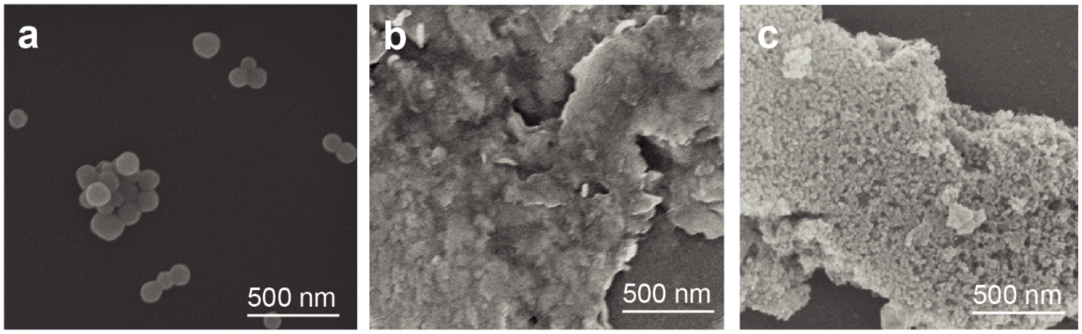

Supplemental Figure 17. SEM images of a silicon wafer after applying a drop of the following solutions and drying them: (**a**) BSA, (**b**) ERM-CA616 groundwater reference and (**c**) Guillard's (F/2) marine water enrichment solution.

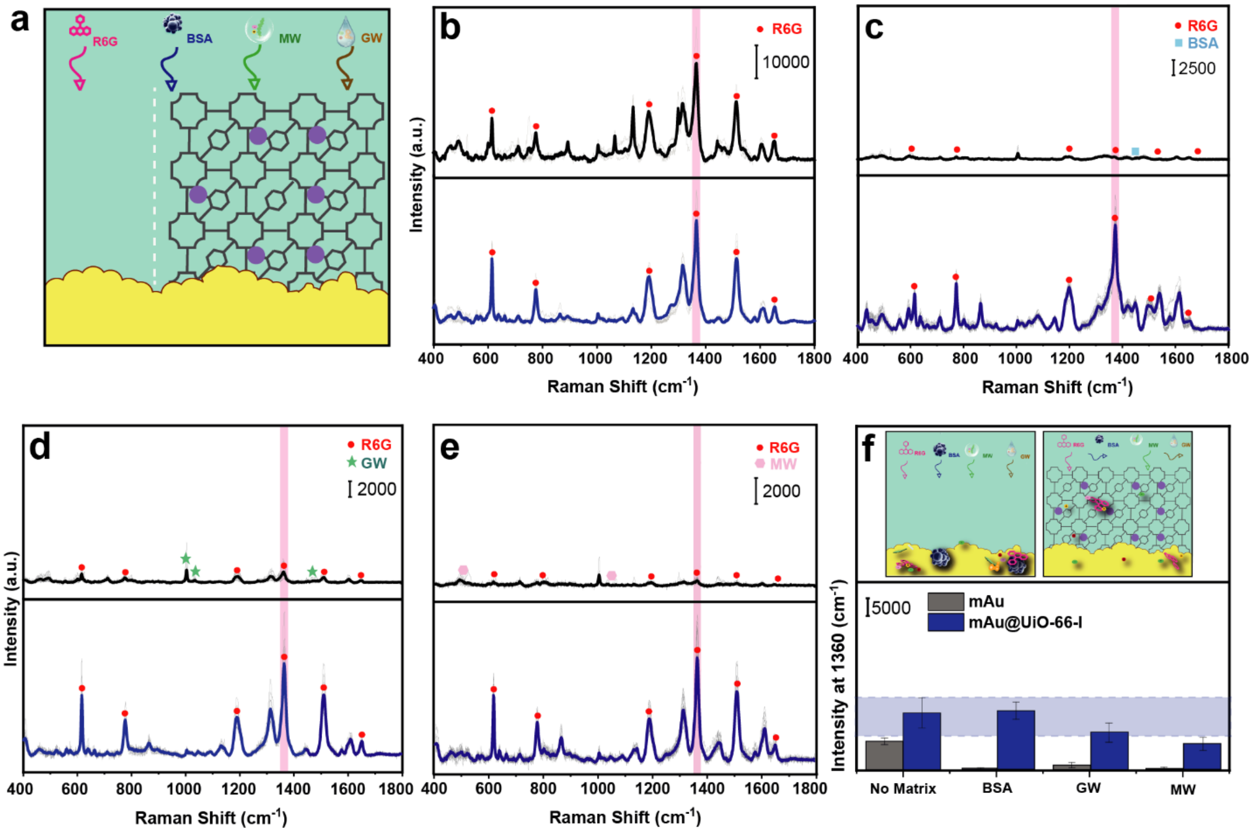

Supplemental Figure 18. SERS sensing of R6G (10^{-4} M) in the presence of interfering components of water: (**a**) common components are bovine serum albumin (BSA), groundwater (GW) and marine water (MW), were mixed with R6G prior to detection. (**b**) SERS spectra of R6G on mAu and mAu@UiO-66-I, mixed with (**c**) BSA, (**d**) GW and (**e**) MW. (**f**) The sensitivities of SERS detection with/without interfering components. (**a**) Schematic illustration showing small R6G molecules (purple) permeate the UiO-66-I layer, whereas large macromolecules like BSA and dissolved ions/organics in groundwater (GW) and marine water (MW) are sterically or chemically rejected. (**b-e**) Raman spectra collected on bare mAu (upper spectra) and on mAu@UiO-66-I (lower spectra). Red circles mark other R6G bands; blue squares, green stars and pink circles denote bands from BSA, GW and MW constituents, respectively. Vertical pink stripe highlights the principal R6G mode at 1360 cm^{-1} used for quantification of (**b**) Neat R6G; (**c**) R6G

+ BSA (10 mg mL^{-1}); (**d**) R6G + GW reference matrix; (**e**) R6G + MW reference matrix. (**f**) Integrated intensity of the 1360 cm^{-1} R6G peak (mean ± s.d., n = 5) for the four test solutions on mAu (grey) and mAu@UiO-66-I (blue). The shaded band corresponds to the R6G signal range obtained without any matrix. The MOF barrier preserves ≥50 % of the R6G signal in all matrices, whereas bare mAu suffers >80 % loss.

Supplemental Note 2 for **Supplemental Figure 18.**

This note clarifies how the *mAu@UiO-66-I* SERS substrate discriminates the small-molecule rhodamine 6G (R6G) from common interferents encountered in environmental matrices (**Supp. Fig. 18a**). The Raman vibrations of R6G are assigned in **Table 5**. R6G has a strong peak at 1360 cm^{-1} with a high signal-to-noise ratio that was used for analysis. Three types of matrices were used:

**Matrix**	**Purpose**	**Reference Table**
Bovine serum albumin (BSA, 10 mg mL^{-1})	Representative high-molecular-weight protein found in wastewater and natural organic matter	**Table 6**
Reference groundwater "GW" (ERM-CA616)	Authentic ionic mixture dominated by Ca^{2+}, Na^{+}, Cl^{-} and orthophosphate; models freshwater.[1]	**Table 7**
Reference marine water "MW" (Sigma G0154)	Concentrate of major nutrients, trace metals and vitamins; models seawater	**Table 8**

Supplemental Figure 18b-e shows the SERS response for mAu (top spectrum) and mAu@UiO-66-I (bottom spectrum) with (**b**) Neat R6G; (**c**) R6G + BSA (10 mg mL^{-1}); (**d**) R6G + GW reference matrix; (**e**) R6G + MW reference matrix. R6G (10^{-4} M in water) was mixed 1:1 (v/v) with each matrix, drop-cast onto either the mAu or mAu@UiO-66-I substrates, then rinsed and measured in SERS. On the mAu substrate the R6G/BSA mixture had a 10X lower R6G intensity compared to mAu@UiO-66-I, accompanied by the appearance of new peaks at 1450 cm^{-1}

corresponding to BSA **(Supp. Fig. 18c**). Analysis of the R6G/BSA mixture on mAu@UiO-66-I showed similar intensity to the spectra without BSA (**Supp. Fig. 18c**). The mAu@UiO-66-I samples showed good sensing performance for R6G in the presense of BSA with no evidence of BSA adsorption in the spectra, confirming the discriminating function of the surface. The mAu@UiO-66-I substrates retained strong R6G signals even in the presence of BSA, while the absence of any BSA bands in the spectra confirms the surface's selective discrimination. Inorganic ions contained in GW and MW interact with the noble metal surface, restricting the target molecule's access to EM hotspots, limiting SERS signal intensity.[2] **Supp. Fig. 18d** shows how GW quenches the R6G response on the bare mAu surface and introduces orthophosphate bands at 1000 cm^{-1} (P-O-C stretching), 1035 cm^{-1} (P=O str), and 1475 cm^{-1} (P=O str), whereas the mAu@UiO-66-I substrate retains strong R6G bands and no obvious contributions from GW. MW contains a concentrate of major nutrients, trace metals and small organic molecules typically found in seawater, which also restrict access to EM hotspots.[3] On bare mAu the R6G bands are attenuated by MW, showing phosphate peaks at 490 cm^{-1} (C-O in-plane deformation), 1033 cm^{-1} and 1062 cm^{-1} (P=O str, C-H in-plane deformation, C-C-C stretching), in addition to biotin and vitamin B12 (**Supp. Fig. 18e**). In contrast, for mAu@UiO-66-I there are no new peaks on the spectra in MW, indicating the blocking of most of the interfering agents. **Supplemental Figure 18f** summarizes the relative intensities of R6G neat in water, BSA, GW and MW. The mAu@UiO66-I samples succeeded in blocking the interference agents and allowing R6G to be detected. However, the R6G signal was attenuated by 17% in GW and 52% in MW, indicating that some inorganic ions are capable of penetrating the UiO-66-I layer. UiO-66 is known to interact with some of these interference agents via electrostatic interactions, which might modulate the final intensity of R6G.[3] The absence of additional peaks suggests that mostly inorganic ions, not

biomolecules, are interfering Raman signal via competitive adsorption, displacing or limiting the adsorption of R6G molecules.

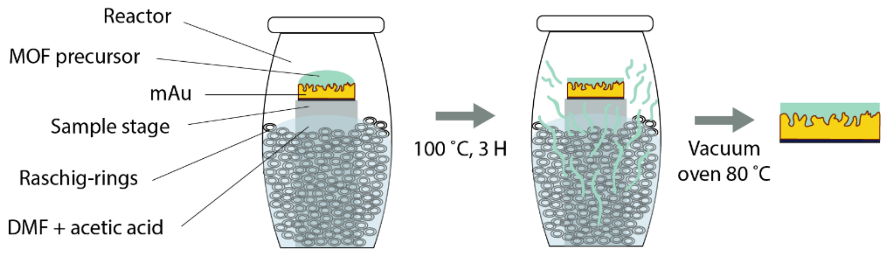

Supplemental Figure 19. The vapour-assisted conversion (VAC) method was used to coat mAu with UiO-66-I. VAC was performed in a 120 mL borosilicate bottle reactor containing Raschig rings and an elevated sample stage. DMF and acetic acid were added to the Raschig rings, then a mAu substrate (5 x 5 mm^2) was placed on the sample stage and the mAu was coated with an 8.8 µL drop of the freshly prepared MOF precursor.

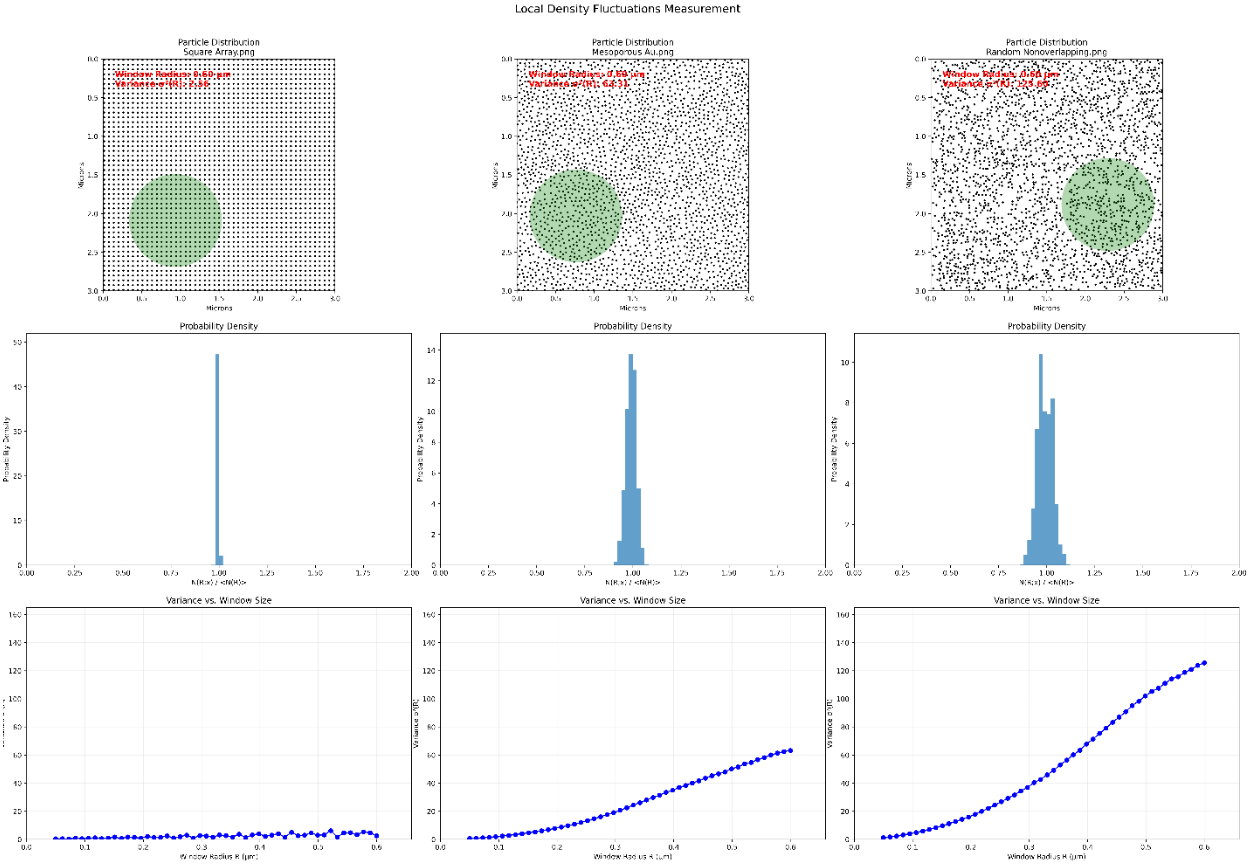

Supplemental Figure 20. Particle distribution maps (**top row**; square array, mesoporous Au, random non-overlapping distribution), probability densities (**middle row**), and window variance (**bottom row**) showing the final frame from **Supplemental Movie 1**.

Supplemental Table 1. Weibull and KS fits for mAu, SQ and RNO samples corresponding to **Figure 1f**.

Sample	Scale Factor (*b*)	Shape Factor (*c*)	KS D	KS p
mAu	11.0 ± 0.991	1.068 ± 0.062	0.069	0.578
SQ	6.14 ± 0.472	1.211 ± 0.083	0.049	0.910
RNO	21.6 ± 3.61	0.613 ± 0.102	0.091	0.293

Supplemental Table 2. Assignment of peaks in the spectra of **Fig. 4a**: mAu@UiO-66-I.

Position (cm^{-1})	Assignation
mAu@UiO-66-I	
289	C-I stretching
434	C-O deformation
485	C-C vibration
497	CC=O deformation, C-O in-plane deformation
624	C-C alicyclic, aliphatic chain vibration
634	C-O-O- stretching, O-C=O in-plane deformation
694	O-C=O in-plane deformation
800	C-C-O stretching, C-O deformation
827	C-I asymmetric stretching, C-C-O stretching
862	Polarised C-C-O stretching
963	=C-H out-of-plane deformation
1087	C=C, polarised C-H in-plane deformation
1143	C-C, C-O-C asymmetric, C-C alicyclic, aliphatic chain vibration
1253	C-H deformation, C-O stretching
1450	O=C-O, C=C in-plane vibration
1616	C=C asymmetric stretch, C=C stretching
1658	C=C stretching

Supplemental Table 3. Assignment of the peaks in the spectra of **Fig. 4a**: 1,4-dichlorobenzene (DCB) with mAu@UiO-66-I.

**Position (cm^{-1})**	**Assignation**
mAu@UiO-66-I+DCB (additional peaks)	
229	C-Cl deformation (new)
305	C-I stretching (new)
419	C-C vibration (DCB)
615	Polarised C-Cl stretching (DCB)
640	C-Cl stretching (new)
702	Polarised C-Cl stretching (DCB)
884	C-H deformation (new)
1001	C-H stretching (DCB)
1034	C-H in-plane deformation (DCB)
1084	C-H in-plane deformation (DCB)
1317	C-H deformation (new)
1593	C-C stretching, C=C stretching (new)

Supplemental Table 4. Assignment of the peaks in the spectra of **Supplemental Fig. 16**: 4-chlorobiphenyl (BiCl) with mAu@UiO-66-I.

**Position (cm^{-1})**	**Assignation**
mAu@UiO66-I+BiCl (additional peaks)	
215	New
287	δ(C-C) aliphatic chains (new)
763	C-C-C bending in-plane (Bi – Cl)
1003	trigonal breathing (Bi – Cl)
1043	C-H bending in-plane (Bi – Cl)
1346	C-H deformation (new)
1558	C=C stretching (new)

Supplemental Table 5. Assignment of the peaks in the spectra of **Supplemental Fig. 18**: R6G with mAu@UiO-66-I.

**Position (cm^{-1})**	**Assignation**
mAu@UiO-66-I+R6G (additional peaks)	
612	C-C-C in-plane bending
775	C-H out-of-plane bending
963	=C-H out-of-plane deformation
1000	C=C, polarized C-H in-plane deformation
1183	C-C stretching
1313	C-H deformation
1365	C=C stretching
1513	C=C stretching
1658	C=C stretching

Supplemental Table 6. Assignment of the Raman peaks of BSA powder (**Fig. 5b**; **Supp. Fig. 18**).

**Peaks (cm^{-1})**	**Assignation**
850	H-bonding of Tyrosin
945	Skeletal C-C stretching
1450	Amide III band

Supplemental Table 7. Composition of certified groundwater "GW" reference material ERM-CA616.

**Mass Concentration**		
	**Certified value [a] [mg/L]**	**Uncertainty [b] [mg/L]**
Calcium	42.6	1.4
Chloride	44.6	0.9
Magnesium	10.1	0.3
Ortho-phosphate	2.24	0.10
Potassium	5.79	0.15
Sodium	27.9	0.8
Ammonium [c]	0.583	-

[a] unweighted mean value of the means of accepted sets of data, each set being obtained in a different laboratory and/or with a different method of determination. The certified values and their uncertainties are traceable to the International System of Units (SI).

[b] The certified uncertainty is the expanded uncertainty estimated in accordance with the Guide to the Expression of Uncertainty in Measurement (GUM, ISO/IEC Guide 98-3:2008) with a coverage factor $k = 2$, corresponding to a level of confidence of about 95 %.

[c] as obtained by ion chromatography.

Supplemental Table 8. Composition of certified marine water "MW" reference material G0154 (Sigma-Aldrich).

Component	Concentration (mg/L)
Biotin	0.0005
Cobalt chloride • $6H_2O$	0.01
Cupric sulfate • $5H_2O$	0.01
EDTA disodium • $2H_2O$	4.36
Ferric chloride • $6H_2O$	3.15
Manganese chloride • $4H_2O$	0.18
Sodium molybdate • $2H_2O$	0.006
Sodium nitrate	75.0
Sodium phosphate monobasic	4.411
Thiamine • HCl	0.1
Vitamin B_{12}	0.0005
Zinc sulfate • $7H_2O$	0.022

Supplemental Table 9. Assignment of the Raman peaks of groundwater "GW" dried on a silicon wafer (**Fig. 5c**; **Supp. Fig. 18**).

Groundwater Peaks (cm^{-1})	Assignation
1000	P-O-C stretching
1035	P=O stretching
1475	P=O stretching

Supplemental Table 10. Assignment of the Raman peaks of marine water "MW" dried on a silicon wafer (**Fig. 5d,e**; **Supp. Fig. 18**).

**Peaks (cm^{-1})**	**Assignation**
490	C-O in-plane deformation, Amide IV Band, C-C=O deformation
1062	C-H in-plane deformation, C-C-C stretching
1320	C-N stretching vibrations, CH_2 wagging modes

Supplemental Table 11. Assignment of the Raman peaks of naphthalene "Napth" (**Fig. 5d,e**)

**Peaks (cm^{-1})**	**Assignation**
512	In-plane vib of benzene
761	C-C stretching
1022	C-H rocking
1385	C-C stretching

References:

1. Yadav, B.; Chavan, S.; Atmakuri, A.; Tyagi, R. D.; Drogui, P. A Review on Recovery of Proteins from Industrial Wastewaters with Special Emphasis on PHA Production Process: Sustainable Circular Bioeconomy Process Development. *Bioresource Technology* **2020**, *317*, 124006.
2. Tezcan, T.; Boyaci, I. H. A New and Facile Route to Prepare Gold Nanoparticle Clusters on Anodic Aluminium Oxide as a SERS Substrate. *Talanta* **2021**, *232*, 122426.
3. Ahmadijokani, F.; Molavi, H.; Rezakazemi, M.; Tajahmadi, S.; Bahi, A.; Ko, F.; Aminabhavi, T. M.; Li, J.-R.; Arjmand, M. UiO-66 Metal-Organic Frameworks in Water Treatment: A Critical Review. *Progress in Materials Science* **2022**, *125*, 100904.
